# Bile Acid–Microbiome Interaction Promotes Gastric Carcinogenesis

**DOI:** 10.1002/advs.202200263

**Published:** 2022-03-14

**Authors:** Shouli Wang, Junliang Kuang, Hongwei Zhang, Wenlian Chen, Xiaojiao Zheng, Jieyi Wang, Fengjie Huang, Kun Ge, Mengci Li, Mingliang Zhao, Cynthia Rajani, Jinshui Zhu, Aihua Zhao, Wei Jia

**Affiliations:** ^1^ Center for Translational Medicine and Shanghai Key Laboratory of Diabetes Mellitus Shanghai Jiao Tong University Affiliated Sixth People's Hospital Shanghai 200233 China; ^2^ Department of Metabolic and Bariatric Surgery Shanghai Jiao Tong University Affiliated Sixth People's Hospital Shanghai 200233 China; ^3^ Cancer Institute, Longhua Hospital Shanghai University of Traditional Chinese Medicine Shanghai 200233 China; ^4^ Cancer Biology Program University of Hawaii Cancer Center Honolulu HI 96813 USA; ^5^ Department of Gastroenterology Shanghai Jiao Tong University Affiliated Sixth People's Hospital Shanghai 200233 China; ^6^ School of Chinese Medicine Hong Kong Baptist University Kowloon Tong Hong Kong 999077 China

**Keywords:** bile acid, bile reflux, gastric carcinogenesis, microbiome

## Abstract

Bile reflux gastritis (BRG) is associated with the development of gastric cancer (GC), but the specific mechanism remains elusive. Here, a comprehensive study is conducted to explore the roles of refluxed bile acids (BAs) and microbiome in gastric carcinogenesis. The results show that conjugated BAs, interleukin 6 (IL‐6), lipopolysaccharide (LPS), and the relative abundance of LPS‐producing bacteria are increased significantly in the gastric juice of both BRG and GC patients. A secondary BA, taurodeoxycholic acid (TDCA), is significantly and positively correlated with the LPS‐producing bacteria in the gastric juice of these patients. TDCA promotes the proliferation of normal gastric epithelial cells (GES‐1) through activation of the IL‐6/JAK1/STAT3 pathway. These results are further verified in two mouse models, one by gavage of TDCA, LPS, and LPS‐producing bacteria (*Prevotella melaninogenica*), respectively, and the other by bile reflux (BR) surgery, mimicking clinical bile refluxing. Moreover, the bile reflux induced gastric precancerous lesions observed in the post BR surgery mice can be prevented by treatment with cryptotanshinone, a plant‐derived STAT3 inhibitor. These results reveal an important underlying mechanism by which bile reflux promotes gastric carcinogenesis and provide an alternative strategy for the prevention of GC associated with BRG.

## Graphical abstract

Bile acids (BAs), especially conjugated BAs, are significantly increased in gastric juice of patients with bile reflux gastritis, which leads to an increase of intragastric pH value and ultimately to a significant increase of lipopolysaccharide‐producing bacteria in the stomach. Bile reflux can promote gastric carcinogenesis through the activation of the IL‐6/JAK1/STAT3 pathway and STAT3 inhibition can alleviate this carcinogenic effect.

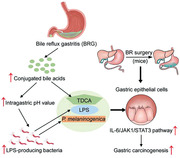

## 1 Introduction

Gastric cancer (GC) is one of the most common and lethal gastrointestinal malignancies worldwide.^[^ ^1^ ^]^ The development of GC, especially the intestinal type, usually occurs through chronic gastric inflammation, atrophic gastritis, and intestinal metaplasia.^[^ ^2^ ^]^ Bile acids (BAs) have been shown to be associated with intestinal metaplasia in the cardia^[^ ^3^, ^4^ ^]^ and bile reflux gastritis (BRG) has been implicated in the genesis of gastritis and gastric cancer in humans and animals.^[^ ^5^, ^6^, ^7^ ^]^ A recent clinical cross‐sectional study showed that bile reflux (BR) is an independent risk factor for the development of precancerous gastric lesions and GC.^[^ ^8^ ^]^ In addition, in the remnant stomach of rats after gastrectomy, BAs, the main component of the duodenal juice, have been implicated in gastric cancer via duodenogastric reflux.^[^ ^9^ ^]^ In humans, duodenogastric reflux has also been implicated in gastric stump carcinoma.^[^ ^10^, ^11^, ^12^ ^]^ Recently, it was reported that acidified BAs enhanced tumor progression and telomerase activity of gastric cancer in vivo^[^ ^13^ ^]^ and that deoxycholic acid induced intestinal metaplasia in gastric epithelial cells.^[^ ^14^, ^15^ ^]^

Reflux of the BAs and intestinal bacteria into the stomach was suggested to induce changes in the microbial composition.^[^ ^16^, ^17^ ^]^ According to the recent reports, eradication of *Helicobacter pylori* (*H. pylori*) cannot completely reverse intestinal metaplasia progression,^[^ ^18^, ^19^ ^]^ although *H. pylori* is considered an important etiological factor in both the precursor event and subsequent GC development.^[^ ^20^, ^21^ ^]^ Hence, there are predisposing factors other than *H*. *pylori* infection that may also play important roles in GC development and progression. Many studies have found that there is a close relationship between BAs and microbiota in gastrointestinal inflammation and carcinogenesis.^[^ ^22^, ^23^ ^]^ However, there are few studies focused on gastric microbiota induced by bile refluxing and their influences on the gastric mucosa injury.

In the present study, we found that elevated conjugated BAs (the BAs are conjugated with glycine or taurine) were positively associated with lipopolysaccharide (LPS)‐producing bacteria in patients with BRG and GC. Through in vitro and in vivo assays, we further investigated the roles of taurodeoxycholic acid (TDCA), LPS, and LPS‐producing bacteria in promoting gastric inflammation and precancerous lesions. Our findings suggest that bile reflux can promote gastric carcinogenesis through the activation of the IL‐6/JAK1/STAT3 pro‐inflammatory signaling pathway and that STAT3 inhibition can alleviate this carcinogenic effect, thus providing an alternative strategy for the prevention of gastric cancer associated with bile reflux.

## 2 Results

### 2.1 The Levels of Conjugated BAs Were Highly Associated with Bile Reflux Gastritis

**Figure 1:**
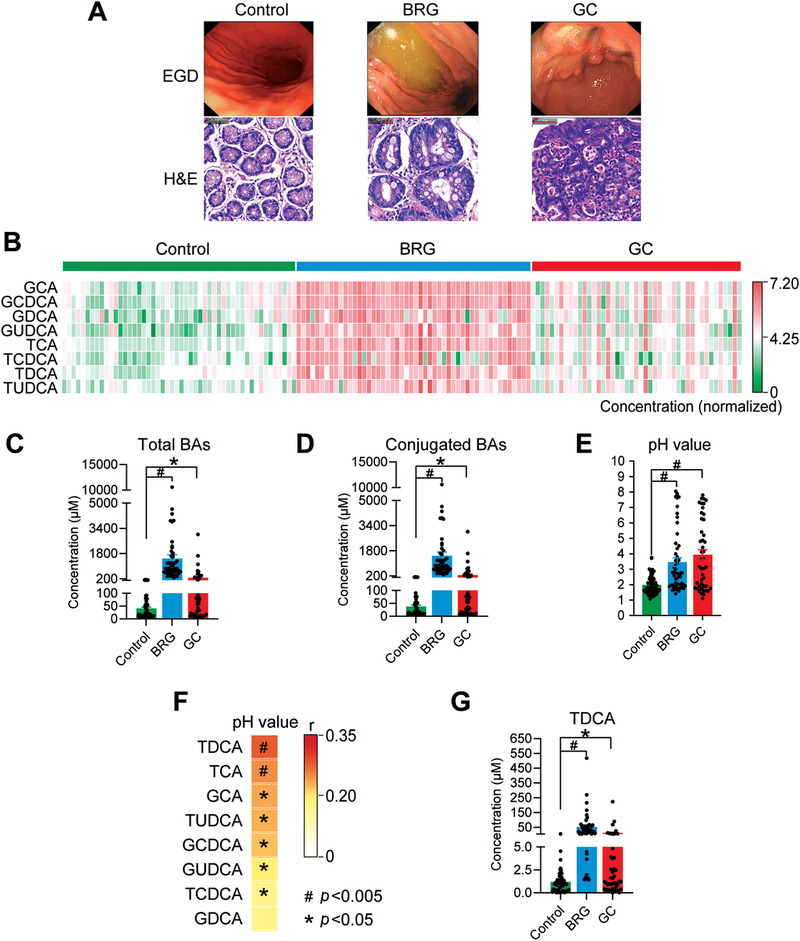
Conjugated BAs were highly associated with bile reflux gastritis. Human gastric sections and juice are analyzed in control (*n* = 50), BRG (*n* = 50), and GC (*n* = 45) groups. A) Representative images of EGD and H&E staining, bars, 50 µm. B) Heatmaps of normalized concentrations of the conjugated BAs in human gastric juice. The color of each cell in the heatmap corresponds to the normalized and log‐transformed raw abundance of the BAs in each sample. C) Concentrations of total BAs in human gastric juice. D) Concentrations of conjugated BAs in human gastric juice. E) pH values of human gastric juice. F) Heatmaps of Spearman correlation coefficients of 8 elevated conjugated BAs and pH value in human gastric juice. The color of the cells indicates the Spearman correlation coefficients (*r*). G) Concentrations of TDCA in human gastric juice. Data are shown as mean with SEM. Differences between groups were assessed using the Kruskal‐Wallis test, #*p* < 0.005, *p < 0.05. BRG: bile reflux gastritis; GC: gastric cancer; EGD: esophagogastroduodenoscopy; H&E: hematoxylin and eosin; GCA: glycocholic acid; GCDCA: glycochenodeoxycholic acid; GDCA: glycodeoxycholic acid; GUDCA: glycoursodeoxycholic acid; TCA: taurocholic acid; TCDCA: taurochenodeoxycholic acid; TDCA: taurodeoxycholic acid; TUDCA: tauroursodeoxycholic acid; BAs: bile acids.

To investigate the BA characteristic of bile reflux gastritis, we collected gastric juice samples of control (*n* = 50), BRG (*n* = 50), and GC (*n* = 45). The esophagogastroduodenoscopy (EGD) examination revealed large amounts of yellow‐green fluid that resided in the gastric cavity of patients with BRG, and the hematoxylin and eosin (H&E) staining of the stomach showed gastric precancerous lesions (intestinal metaplasia) in the BRG group and gastric cancer cell infiltration in GC group (**Figure** **1**A). The BA profiles of the gastric juice samples among control, BRG, and GC groups were measured using an ultra‐performance liquid chromatography coupled with a triple quadrupole mass spectrometry (UPLC/TQMS) based targeted metabolomics approach. The results revealed that the total BAs (the sum of all detected conjugated and unconjugated BAs (the BAs in the free form)), especially conjugated BAs, were significantly elevated in patients with BRG and GC (Figure 1B–D), among which glycocholic acid (GCA), glycochenodeoxycholic acid (GCDCA), glycodeoxycholic acid (GDCA), glycoursodeoxycholic acid (GUDCA), taurocholic acid (TCA), taurochenodeoxycholic acid (TCDCA), TDCA, and tauroursodeoxycholic acid (TUDCA) increased substantially (Figure S1A, Supporting Information). Meanwhile, the unconjugated BAs in the gastric juice of the BRG and GC groups also increased significantly (Figure S1B, Supporting Information), but the absolute concentrations and the fold change (FC) of them were much lower (FC = 1.46 for BRG/control, FC = 1.61 for GC/control) than those of conjugated BAs (FC = 39.10 for BRG/control, FC = 6.62 for GC/control).

The increased BAs led us to consider whether the inherent acid‐base balance in the stomach was disturbed. We then measured the pH value of gastric juice and the results showed that pH increased drastically in patients with BRG (3.46 ± 0.28) and GC (3.93 ± 0.33) compared with control (1.98 ± 0.09) (Figure 1E). Compared with the strong acid in the stomach, the bile acids refluxing into the stomach from the duodenum are weak acids, which can break the original acid–base balance and increase the pH value in the stomach. We further identified a strong and positive correlation between 7 out of 8 conjugated BAs and the pH value of human gastric juice, in which TDCA showed the strongest positive correlation with pH value (Figure 1F). Meanwhile, the levels of TDCA were significantly increased in the gastric juice of BRG and GC groups (Figure 1G).

Together, these results suggest that there were significantly elevated levels of conjugated BAs in the gastric juice of BRG and GC patients, leading to the increase of gastric pH value and probably inducing precancerous lesions.

### 2.2 Increased Conjugated BAs Were Associated with the Elevated Abundance of LPS‐Producing Bacteria

**Figure 2:**
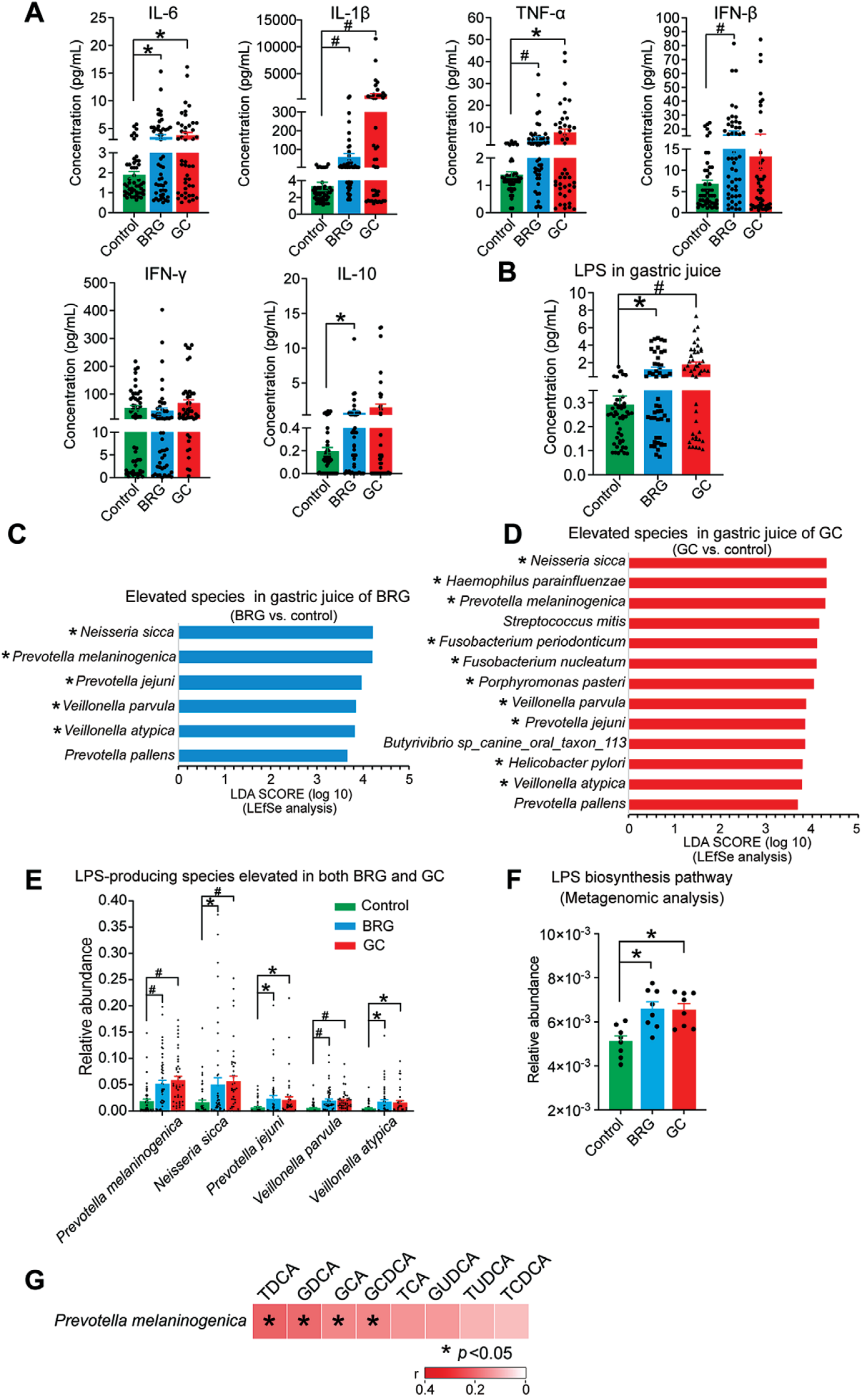
Increased conjugated BAs were associated with the elevated abundance of LPS‐producing bacteria. A) Concentrations of inflammatory cytokines in human gastric juice from control, BRG, and GC groups. B) Concentrations of LPS in human gastric juice from control, BRG, and GC groups. C) Elevated species in gastric juice of BRG group based on the LEfSe analysis between BRG and control groups. The species marked with asterisks are those with LPS biosynthesis function in the metagenomic analysis. D) Elevated species in gastric juice of GC group based on the LEfSe analysis between GC and control groups. The species marked with asterisks are those with LPS biosynthesis function in the metagenomic analysis. E) Relative abundance of the 5 LPS‐producing species elevated in both BRG and GC groups. F) Relative abundance of the LPS biosynthesis pathway in human gastric microbiota based on metagenomic analysis. G) Heatmaps of Spearman correlation coefficients of 8 elevated conjugated BAs and *Prevotella melaninogenica* in human gastric juice. The color of the cells indicates the Spearman correlation coefficients (*r*). Data are shown as mean with SEM. Differences between groups were assessed using the Kruskal‐Wallis test, #*p* < 0.005, *p < 0.05. BRG: bile reflux gastritis; GC: gastric cancer; LEfSe: linear discriminant analysis effect size; LPS: lipopolysaccharide; TDCA: taurodeoxycholic acid; GDCA: glycodeoxycholic acid; GCA: glycocholic acid; GCDCA: glycochenodeoxycholic acid; TCA: taurocholic acid; GUDCA: glycoursodeoxycholic acid; TUDCA: tauroursodeoxycholic acid; TCDCA: taurochenodeoxycholic acid.

According to the reports, the BAs refluxing into the stomach were closely related to chronic inflammation.^[^ ^24^, ^25^ ^]^ So, we measured pro‐inflammatory cytokines in gastric juice and the results showed that compared with control group, the concentrations of IL‐6, interleukin‐1*β* (IL‐1*β*), and tumor necrosis factor alpha (TNF‐*α*) were significantly increased in the BRG and GC groups (**Figure** **2**A). We also found that the concentrations of LPS in gastric juice were significantly increased in the BRG and GC groups but no difference of LPS levels was detected between BRG and GC groups (Figure 2B).

To further determine whether the levels of LPS‐producing bacteria increased correspondingly, we assessed microbial diversity and richness of gastric juice samples via the analysis of full‐length 16S rRNA gene sequencing. By measuring *α* diversity using the Shannon index, we found that there was no significant difference among the three groups. Next, we determined the significantly increased species in the BRG (Figure 2C) and GC (Figure 2D) groups by linear discriminant analysis effect size (LEfSe) differential analysis, compared to the control group. The relative abundance of *Neisseria sicca* (*N. sicca*)*, Prevotella melaninogenica* (*P. melaninogenica*), *P. jejuni*, *Veillonella parvula* (*V. parvula*), *V. atypica*, and *P. pallens* were significantly elevated in both BRG and GC groups with *P. melaninogenica* having the highest relative abundance in both BRG and GC groups, 5.15% and 5.88%, respectively (Figure 2E). We next applied a metagenomic approach and found that most of the species significantly increased in gastric juice of BRG (5 of 6, 83%) and GC (10 of 13, 77%) groups were LPS‐producing bacteria (Figure 2C,D, marked with asterisks), consistent with the significant increase of LPS levels (Figure 2B). However, none of the five elevated species in the control group was LPS‐producing bacteria (Figure S1C, Supporting Information). In addition, the pathway analysis of metagenomic data showed that the activation of the LPS biosynthetic pathway was significantly enriched in both BRG and GC groups compared with control group (Figure 2F). In order to explore the relationship between the refluxed BAs and LPS‐producing bacteria, we performed Spearman correlation analysis and found that except *N. sicca*, the other four LPS‐producing bacteria had a significant positive correlation with the elevated conjugated BAs (Figure 2G; Figure S1D, Supporting Information), in which TDCA had the strongest positive correlation with *P. melaninogenica* (Figure 2G).

Collectively, our results demonstrated that bile reflux may have induced significant changes in the composition of gastric microbiota, mainly manifested as an increase in the relative abundance of LPS‐producing bacteria, a result consistent with the observed increased amounts of LPS in gastric juice.

### 2.3 TDCA and LPS Promoted Growth of Gastric Epithelial Cells

**Figure 3:**
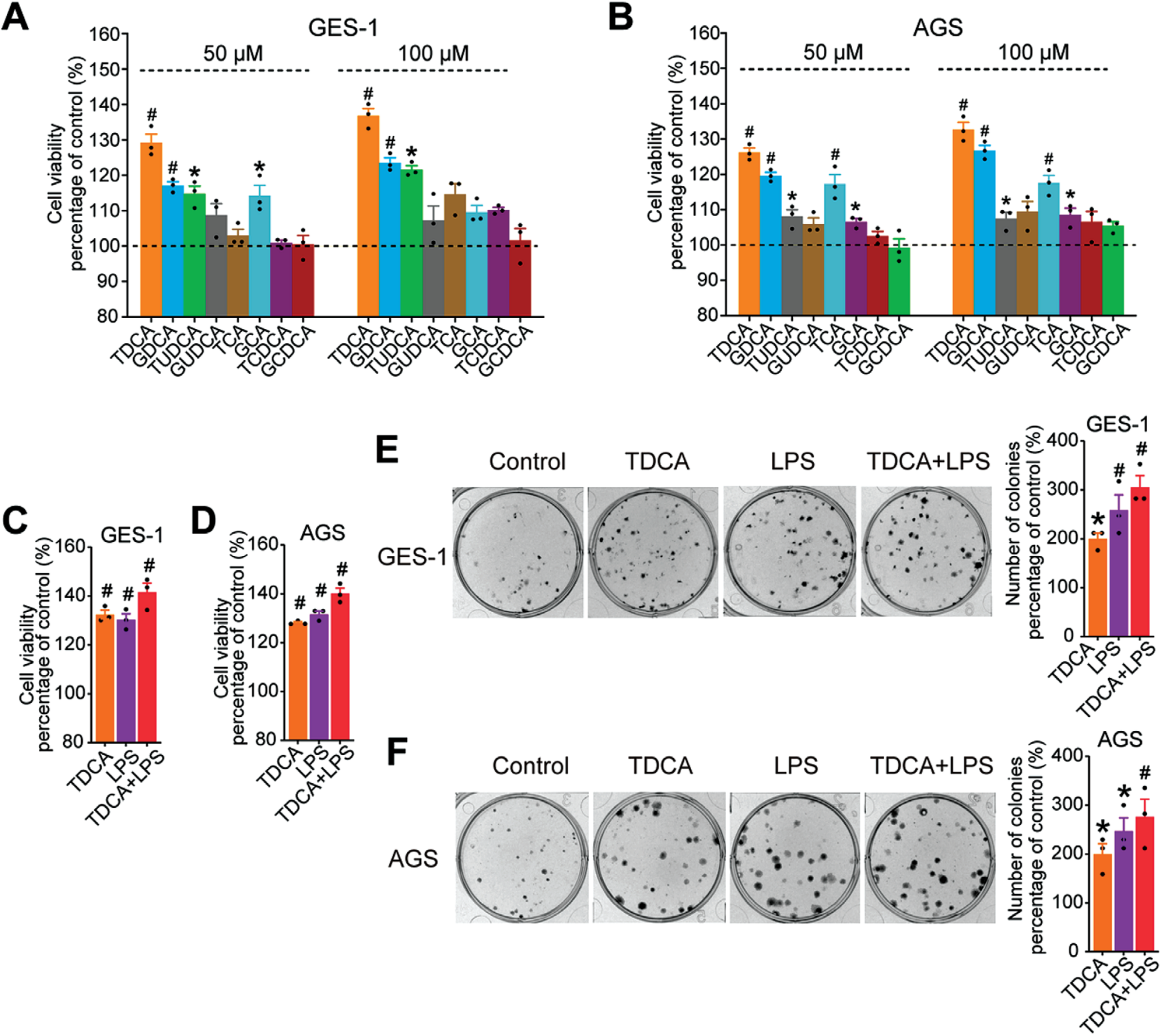
TDCA and LPS promoted the growth of gastric epithelial cells. A) The effects of 8 conjugated BAs on regulating cell proliferation of normal gastric epithelial cells (GES‐1) were evaluated by a Cell Counting Kit‐8 (CCK‐8) assay. B) The effects of 8 conjugated BAs on regulating cell proliferation of gastric cancer cell line (AGS) were evaluated by a CCK‐8 assay. C) TDCA, LPS, and TDCA+LPS significantly increased cell proliferation of GES‐1. D) TDCA, LPS, and TDCA+LPS significantly increased cell proliferation of AGS. E) TDCA, LPS, and TDCA+LPS significantly increased colony formation of GES‐1. F) TDCA, LPS, and TDCA+LPS significantly increased colony formation of AGS. Data are shown as mean with SEM. Differences between groups (all groups compared to the control group) were assessed using the one‐way ANOVA test, #*p* < 0.005, *p < 0.05. TDCA: taurodeoxycholic acid; GDCA: glycodeoxycholic acid; TUDCA: tauroursodeoxycholic acid; GUDCA: glycoursodeoxycholic acid; TCA: taurocholic acid; GCA: glycocholic acid; TCDCA: taurochenodeoxycholic acid; GCDCA: glycochenodeoxycholic acid; LPS: lipopolysaccharide.

To verify whether the conjugated BAs and LPS influence the growth of gastric epithelial cells in vitro, we performed cell proliferation assays to determine the effects of the conjugated BAs (0, 50, 100 µm final concentrations) and LPS (0, 50, 100 ng mL^−1^ final concentrations) on the growth of the normal gastric epithelial cell line (GES‐1) (**Figure** **3**A) and the gastric cancer cell line (AGS) (Figure 3B). The results showed that TDCA had the greatest effect on promoting cell growth of both GES‐1 and AGS in a dose‐dependent manner. We then used TDCA (100 µm), LPS (100 ng mL^−1^), and TDCA (100 µm) with LPS (100 ng mL^−1^) to treat both GES‐1 and AGS cells. The results showed that the TDCA with LPS treated group exhibited a higher proliferation rate (Figure 3C,D). Consistent with the results of proliferation assays, the number of clones of GES‐1 (Figure 3E) and AGS (Figure 3F) increased significantly after TDCA (100 µm), LPS (100 ng mL^−1^), and TDCA (100 µm) with LPS (100 ng mL^−1^) respective interventions. These results revealed that TDCA with LPS promoted the most cell growth of GES‐1 and AGS cells.

To further investigate how TDCA and LPS directly promote the cell proliferation, we performed the Western blot analysis of the proliferation markers, Ki‐67 and proliferating cell nuclear antigen (PCNA) in the GES‐1 cells with TDCA (100 µm), LPS (100 ng mL^−1^), and TDCA (100 µm) with LPS (100 ng mL^−1^) intervention for 24 h. The results showed that the protein levels of Ki‐67 were significantly increased after treatment with TDCA, LPS, and TDCA with LPS, while no significant changes were found in PCNA (Figure S2, Supporting Information). Furthermore, we found the protein levels of Cyclin D1, a regulator of cell cycle progression, were significantly upregulated by TDCA, LPS, and TDCA with LPS (Figure S2, Supporting Information), which suggested that TDCA and LPS may promote cell proliferation by accelerating cell cycle.

### 2.4 TDCA and LPS Promoted Gastric Inflammation in Mice

**Figure 4:**
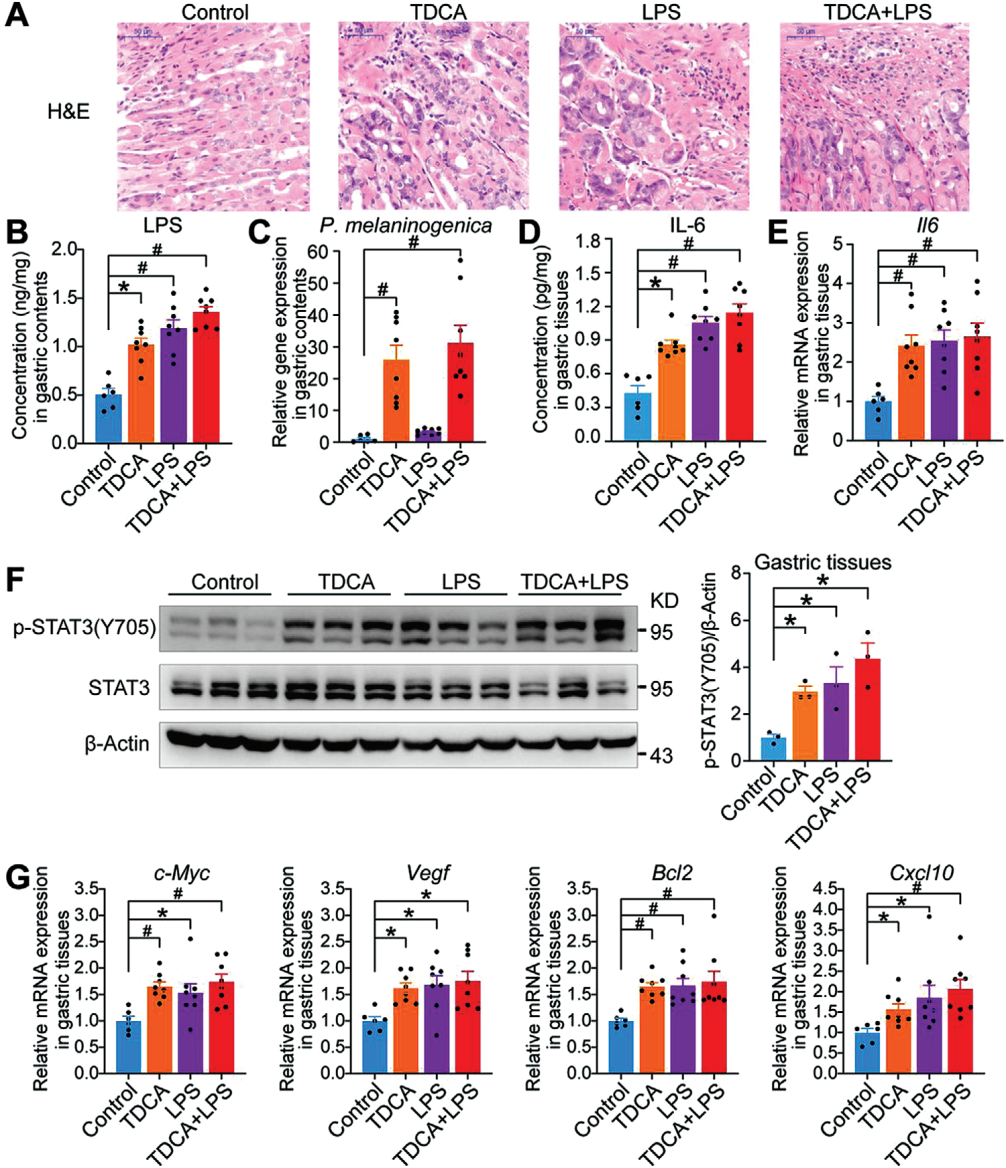
TDCA and LPS promoted gastric inflammation in mice. A) Representative images of H&E staining of gastric tissues from control (*n* = 6), TDCA (*n* = 8), LPS (*n* = 8), and TDCA+LPS (*n* = 8) groups, bars, 50 µm. B) Concentrations of LPS in gastric contents from control, TDCA, LPS, and TDCA+LPS groups. C) Relative gene expression of *P. melaninogenica* in gastric contents from control, TDCA, LPS, and TDCA+LPS groups by RT‐qPCR. D) Concentrations of IL‐6 in gastric tissues from control, TDCA, LPS, and TDCA+LPS groups. E) Relative mRNA expression of *Il6* in gastric tissues from control, TDCA, LPS, and TDCA+LPS groups. F) The gastric protein expression of STAT3 in the mice from control, TDCA, LPS, and TDCA+LPS groups. G) Relative mRNA expression of STAT3 target genes in gastric tissues from control, TDCA, LPS, and TDCA+LPS groups. Data are shown as mean with SEM. Differences between groups were assessed using the one‐way ANOVA test or Kruskal‐Wallis test, #*p* < 0.005, *p < 0.05. H&E: hematoxylin and eosin; TDCA: taurodeoxycholic acid; LPS: lipopolysaccharide.

Our clinical results showed that bile reflux can significantly increase the levels of inflammatory factors. To further investigate whether TDCA and LPS can promote gastric inflammation in vivo, we used saline (control vehicle), TDCA (120 mg/kg/day), LPS (0.05 mg/kg/day), and TDCA (120 mg/kg/day) with LPS (0.05 mg/kg/day) to treat C57BL/6J mice for 43 weeks. During 43 weeks of intervention, there were only slight differences in the mice body weights (Figure S3A, Supporting Information) and food intake (Figure S3B, Supporting Information). Obvious inflammatory cell infiltration in gastric tissues was found in mice treated with 43 weeks of TDCA, LPS, and TDCA with LPS (**Figure** **4**A), suggesting that long‐term intervention of TDCA and LPS promoted gastric inflammation in mice. Moreover, levels of LPS and LPS‐producing bacteria (*P. melaninogenica*) were significantly increased in gastric contents of TDCA, LPS, and TDCA with LPS groups (Figure 4B,C), which was consistent with our clinical findings. The concentrations of TDCA were significantly increased in the contents and tissues of the TDCA and TDCA with LPS groups (Figure S3C–J, Supporting Information). These results provided supporting evidence that TDCA and LPS were capable of promoting gastric inflammation in mice.

To assess whether TDCA and LPS induced the increase of IL‐6 level, we performed enzyme‐linked immunosorbent assay (ELISA) and quantitative real‐time polymerase chain reaction (RT‐qPCR) and the results showed that both IL‐6 concentrations and gene expression were significantly increased in TDCA, LPS, and TDCA with LPS treated groups, with the highest level in TDCA with LPS group (Figure 4D,E). Activation of glycoprotein 130 (GP130), also known as IL‐6 signal transducer, promotes the phosphorylation of Janus kinase 1 (JAK1), which in turn phosphorylates signal transducer and activator of transcription 3 (STAT3) on tyrosine‐705 (p‐STAT3 (Y705)). Constitutive activation of STAT3 protein is a common pro‐inflammatory oncogenic feature identified in numerous solid tumors including gastric cancer.^[^ ^26^, ^27^ ^]^ To confirm if the increase of IL‐6 levels induced by TDCA and LPS can activate the STAT3 pathway, we examined the levels of STAT3 phosphorylation using Western blot analysis and immunohistochemistry staining. The results showed that the levels of STAT3 phosphorylation were significantly increased in TDCA, LPS, and TDCA with LPS groups (Figure 4F; Figure S4, Supporting Information). Furthermore, the mRNA of the STAT3 target genes (*c‐Myc, Vegf, Bcl2 and Cxcl10*) were upregulated correspondingly (Figure 4G). These results suggested that TDCA and LPS promoted gastric inflammation in mice, probably through the activation of STAT3 signaling pathway. In addition, the immunohistochemistry results showed that the protein levels of Ki‐67 were significantly increased in the TDCA, LPS, and TDCA with LPS groups (Figure S5, Supporting Information).

### 2.5 *P. melaninogeni*ca Induced Gastric Inflammation in Mice

**Figure 5:**
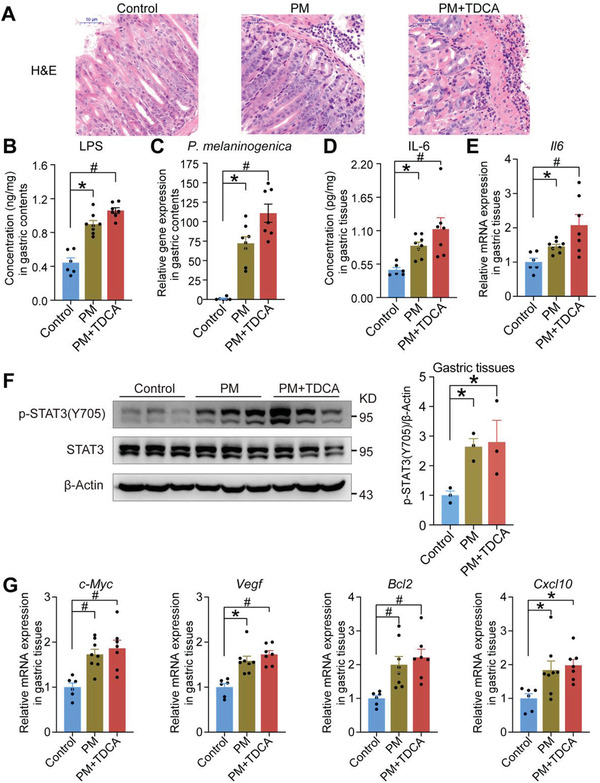
*P. melaninogenica* induced gastric inflammation in mice. A) Representative images of H&E staining of gastric tissues from control (*n* = 6), PM (*n* = 8), and PM+TDCA (*n* = 7) groups, bars, 50 µm. B) Concentrations of LPS in gastric contents from control, PM, and PM+TDCA groups. C) Relative gene expression of *P. melaninogenica* in gastric contents from control, PM, and PM+TDCA groups by RT‐qPCR. D) Concentrations of IL‐6 in gastric tissues from control, PM, and PM+TDCA groups. E) Relative mRNA expression of *Il6* in gastric tissues from control, PM, and PM+TDCA groups. F) The gastric protein expression of STAT3 in the mice from control, PM, and PM+TDCA groups. G) Relative mRNA expression of STAT3 target genes in gastric tissues from control, PM, and PM+TDCA groups. Data are shown as mean with SEM. Differences between groups were assessed using the one‐way ANOVA test or Kruskal‐Wallis test, #*p* < 0.005, *p < 0.05. H&E: hematoxylin and eosin; PM: *P. melaninogenica*; TDCA: taurodeoxycholic acid; LPS: lipopolysaccharide.

To verify whether LPS‐producing bacteria can promote gastric inflammation, we used *P. melaninogenica* (PM) (1 × 10^9^ cfu/day) and PM (1 × 10^9^ cfu/day) with TDCA (120 mg/kg/day) to treat C57BL/6J mice for 43 weeks, and saline for the control group. Body weights (Figure S6A, Supporting Information) and food intakes (Figure S6B, Supporting Information) had slight changes between control, PM, and PM with TDCA groups. Compared with the control group, there was obvious inflammatory cell infiltration in gastric tissues of PM and PM with TDCA groups (**Figure** **5**A). Accordingly, the levels of LPS and LPS‐producing bacteria, *P. melaninogenica* in gastric contents were substantially elevated in PM and PM with TDCA groups, in which PM with TDCA group had a higher level (Figure 5B,C). The concentrations of TDCA in gastric contents and tissues were significantly increased in PM with TDCA group (Figure S6C–J, Supporting Information). Meanwhile, IL‐6 concentration and gene expression showed similar group differences (Figure 5D,E). The results of both Western blot analysis and immunohistochemistry staining showed that the levels of STAT3 phosphorylation were significantly increased in PM and PM with TDCA groups (Figure 5F; Figure S7, Supporting Information). Additionally, the mRNA levels of STAT3 target genes were also significantly upregulated in the two interventions groups (Figure 5G). Taken together, LPS‐producing bacteria, *P. melaninogenica*, promoted gastric inflammation in mice and activation of the STAT3 signaling pathway. The immunohistochemistry results showed that the protein levels of Ki‐67 were significantly increased in the PM and PM with TDCA groups (Figure S8, Supporting Information).

### 2.6 TDCA and LPS Promoted Gastric Carcinogenesis by Activating IL‐6/JAK1/STAT3 Pathway in Gastric Epithelial Cells

**Figure 6:**
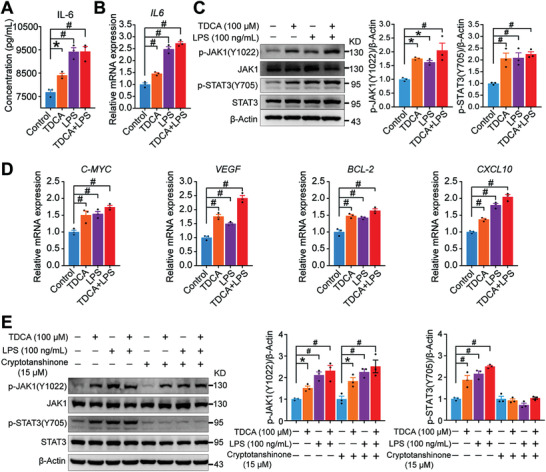
TDCA and LPS promoted gastric carcinogenesis by activating IL‐6/JAK1/STAT3 pathway in gastric epithelial cells. A) Concentrations of IL‐6 in the supernatant of GES‐1 cells increased significantly in TDCA, LPS, and TDCA+LPS groups. B) Relative mRNA expression of *IL6* in GES‐1 cells increased significantly in TDCA, LPS, and TDCA+LPS groups. C) Western blot analysis showing the activation of IL‐6/JAK1/STAT3 pathway in TDCA, LPS, and TDCA+LPS groups. D) Relative mRNA expression of STAT3 target genes in GES‐1 cells increased significantly in TDCA, LPS, and TDCA+LPS groups. E) Western Blot results showed STAT3 inhibitor (cryptotanshinone) markedly attenuated the activation effect of IL‐6/JAK1/STAT3 pathway in GES‐1 cells. Data are shown as mean with SEM. Differences between groups were assessed using the one‐way ANOVA test, #*p* < 0.005, *p < 0.05. TDCA: taurodeoxycholic acid; LPS: lipopolysaccharide.

To determine whether TDCA and LPS can also promote the activation of IL‐6 and STAT3 pathways in vitro, we used TDCA (100 µm), LPS (100 ng mL^−1^), and TDCA (100 µm) with LPS (100 ng mL^−1^) to intervene GES‐1 cells for 4 h. We found that both the concentrations of IL‐6 in the supernatant and the mRNA expression levels were significantly increased after treatment with TDCA, LPS, and TDCA with LPS (**Figure** **6**A,B), which were consistent with the results of our clinical and in vivo experiments (Figures 2A, 4D, and 5D). These results suggested that TDCA and LPS promote the secretion of IL‐6 in GES‐1 cells.

To examine whether the increase in IL‐6 levels induced by TDCA and LPS can activate the STAT3 pathway, GES‐1 cells were treated with TDCA (100 µm), LPS (100 ng mL^−1^), and TDCA (100 µm) with LPS (100 ng mL^−1^) for 24 h. The results indicated that both TDCA and LPS activated the phosphorylation of JAK1(Y1022), STAT3 (Y705), and the mRNA expression levels of STAT3 target genes (*VEGF*, *C‐MYC*, *BCL‐2*, and *CXCL10*) (Figure 6C,D). These results suggested that TDCA and LPS promoted the growth of gastric epithelial cells possibly through the activation of the IL‐6/JAK1/STAT3 pathway.

To further verify the effects of TDCA and LPS on STAT3 activation, GES‐1 cells were treated with an inhibitor of STAT3, cryptotanshinone (15 µm) for 30 min before the 24‐h intervention with TDCA (100 µm), LPS (100 ng mL^−1^), and TDCA (100 µm) with LPS (100 ng mL^−1^). Cryptotanshinone is a bioactive compound isolated from the roots of *Salvia miltiorrhiza* that specifically blocks STAT3 phosphorylation at Tyr705.^[^ ^28^ ^]^ The results showed that the effects of TDCA and LPS on activating STAT3 were abolished in the presence of cryptotanshinone (Figure 6E), supporting our hypothesis that TDCA and LPS promote the proliferation of gastric epithelial cells by IL‐6/JAK1/STAT3 signaling.

### 2.7 Bile Reflux Caused Gastric Carcinogenesis and Cryptotanshinone Achieved Targeted Therapeutic Effects in Mice

**Figure 7:**
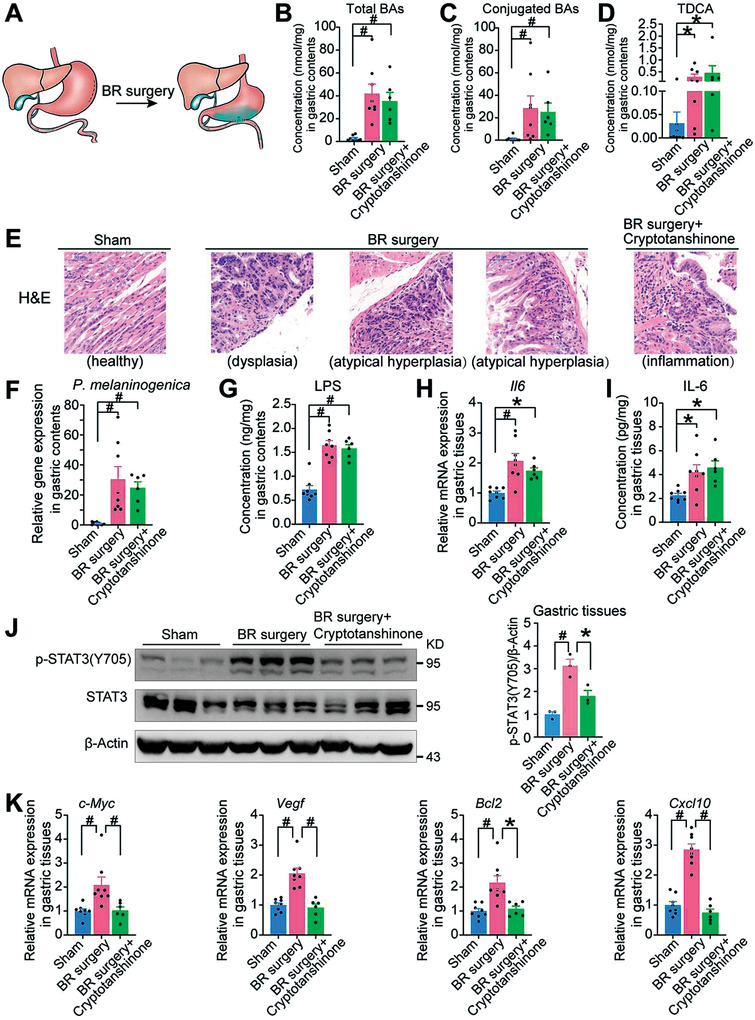
Bile reflux caused gastric carcinogenesis and cryptotanshinone achieved targeted therapeutic effects in mice. A) Schematic diagram of the BR surgery in C57BL/6J mice. B) Concentrations of total BAs in gastric contents from sham (*n* = 8), BR surgery (*n* = 8), and BR surgery+cryptotanshinone (*n* = 6) groups. C) Concentrations of conjugated BAs in gastric contents from sham, BR surgery, and BR surgery+cryptotanshinone groups. D) Concentrations of TDCA in gastric contents from sham, BR surgery, and BR surgery+cryptotanshinone groups. E) Representative images of H&E staining of gastric tissues from sham, BR surgery, and BR surgery+cryptotanshinone groups, bars, 50 µm. F) Relative gene expression of *P. melaninogenica* in gastric contents from sham, BR surgery, and BR surgery+cryptotanshinone groups by RT‐qPCR. G) Concentrations of LPS in gastric contents from sham, BR surgery, and BR surgery+cryptotanshinone groups. H) Relative mRNA expression of *Il6* in gastric tissues from sham, BR surgery, and BR surgery+cryptotanshinone groups. I) Concentrations of IL‐6 in gastric tissues from sham, BR surgery, and BR surgery+cryptotanshinone groups. J) The gastric protein expression of STAT3 in the mice from sham, BR surgery, and BR surgery+cryptotanshinone groups. K) Relative mRNA expression of STAT3 target genes in gastric tissues from sham, BR surgery, and BR surgery+cryptotanshinone groups. Data are shown as mean with SEM. Differences between groups were assessed using the one‐way ANOVA test or Kruskal‐Wallis test, #*p* < 0.005, *p < 0.05. BR: bile reflux; LPS: lipopolysaccharide; TDCA: taurodeoxycholic acid; H&E: hematoxylin and eosin.

To further approach the effects of clinical BRG on gastric carcinogenesis, we conducted a BR surgery model of C57BL/6J mice. Briefly, side‐to‐side anastomosis was performed between the greater curvature of the stomach and the upper jejunum located approximately 1.5 cm distally from the pylorus ring (**Figure** **7**A). Mice in the BR surgery with cryptotanshinone group received intraperitoneal injections of cryptotanshinone. For 50 weeks after surgery, all mice were fasted overnight before being euthanized. Overall, the body weights and food intakes of the sham group had slight differences relative to the BR surgery and BR surgery with cryptotanshinone groups, which indicated BR surgery had no significant effects on the body weights (Figure S9A, Supporting Information) and slight changes on food intakes of mice (Figure S9B, Supporting Information). As expected, the total BAs, conjugated BAs, and TDCA in gastric contents were greatly increased in the BR surgery and BR surgery with cryptotanshinone groups, which were more than ten times of that in the sham group (Figure 7B–D). These results indicated that we successfully established a bile reflux mouse model through the BR surgery. In addition, total BAs, conjugated BAs, and TDCA concentrations had a similar group difference in gastric tissues (Figure S9C–H, Supporting Information).

The results of H&E showed that 50 weeks after BR surgical intervention, 3 out of 8 mice in the BR surgery group developed gastric lesions, of which 1 was a precancerous lesion (dysplasia), and the remaining 2 were atypical hyperplasia (Figure 7E). However, the 6 mice in the BR surgery with cryptotanshinone group showed no obvious gastric lesions, only inflammatory cell infiltration, which suggested that cryptotanshinone has a potential therapeutic effect on gastric injury caused by bile reflux in mice. These results demonstrated that bile reflux promoted gastric carcinogenesis in mice which could be markedly attenuated by the STAT3 inhibitor, cryptotanshinone.

Consistent with our clinical study, the level of *P. melaninogenica* was greatly increased in the gastric contents of the BR surgery and BR surgery with cryptotanshinone groups (Figure 7F), which implied that BR surgery promoted the growth of *P. melaninogenica*. Accordingly, the LPS level in gastric contents had similar group differences (Figure 7G). Meanwhile, both the IL‐6 concentrations and mRNA expression were significantly increased in the BR surgery and BR surgery with cryptotanshinone groups (Figure 7H,I). Both the results of Western blot analysis and immunohistochemistry staining showed that the phosphorylation levels of STAT3 significantly increased in the BR surgery group compared to the sham group (Figure 7J; Figure S10, Supporting Information). However, when the BR surgery with cryptotanshinone group was compared with the BR surgery group, the level of STAT3 phosphorylation was significantly decreased, indicating that cryptotanshinone had an offsetting effect on the activation of STAT3 induced by bile reflux. The RT‐qPCR results revealed similar group differences for the mRNA expression of STAT3 target genes (Figure 7K). Consistent with the previous cell and animal results, BR surgery induced an increase in the protein levels of Ki‐67 compared with the sham group (Figure S11, Supporting Information). However, when the BR surgery with cryptotanshinone group was compared with the BR surgery group, the levels of Ki‐67 were obviously decreased (Figure S11, Supporting Information).

## 3 Discussion

Bile reflux was found to be closely associated with the development of gastric precancerous lesions and gastric cancer,^[^ ^29^, ^30^, ^31^ ^]^ but the reason for this connection remains unclear. The length of time for BAs exposure correlated with the severity of pathological changes in the gastric mucosa.^[^ ^24^ ^]^ In addition, BAs directly induced intestinal metaplasia and progression to neoplasia of the stomach.^[^ ^29^, ^32^, ^33^ ^]^ Although BAs are thought to be critical in the pathogenesis of gastric mucosal diseases, the mechanisms by which BAs induce transformation in the stomach are not yet clear.^[^ ^34^ ^]^ Previously, some researchers used deoxycholic acid to establish chronic gastritis animal models.^[^ ^35^ ^]^ Other previous studies proposed that bile reflux functioned as an initiator of gastric carcinogenesis.^[^ ^4^, ^36^ ^]^

In this study, we have shown that BAs especially conjugated BAs were significantly increased in gastric juice of patients with BRG and GC. The increased BAs were found to be associated with the elevated abundance of LPS‐producing bacteria. Meanwhile, the levels of LPS and IL‐6 in gastric juice also increased significantly in BRG and GC groups. Further, we provided multiple lines of evidence, using in vitro and in vivo models that supported a role for TDCA and LPS in the upregulation of IL‐6/JAK1/STAT3 pathway activity. Finally, we established a BR model in mice by gastrojejunostomy, and verified the results found in our clinical study.

In previous studies, deoxycholic acid or chenodeoxycholic acid was commonly selected directly for animal or cell experiments to explore the effect of bile reflux on gastric cancer,^[^ ^14^, ^37^, ^38^ ^]^ however, the rationality for BAs selection was not discussed. Our study identified specifically that conjugated BAs were increased in the BRG and GC groups with only minimal changes in unconjugated BAs. In addition, the concentrations of conjugated BAs were much higher than that of unconjugated BAs. Therefore, we used the significantly elevated TDCA in the BRG group as an intervention in our cell and animal experiments to be consistent with the clinical findings.

Many existing studies have focused on the effect of BAs on the gastric mucosa after BRG,^[^ ^13^, ^39^, ^40^ ^]^ but less attention has been paid to the effect of significantly changed intragastric bacteria, which may play an important role in the development of gastric mucosa lesions. It was generally perceived that microbial colonization of the stomach was unlikely since gastric conditions are inhospitable to microorganisms.^[^ ^41^ ^]^ In the present study, increased conjugated BAs led to a decreased gastric acidity and thus, we hypothesized that the microbiota composition had significantly changed in the stomach of patients with BRG and GC. Using full‐length 16S rRNA gene sequences analysis, we found that the bacterial community structure in the BRG and GC groups changed significantly. The most significant change was an increase in the relative abundance of LPS‐producing bacteria, such as *P. melaninogenica* and *P. jejuni*. Furthermore, we applied a metagenomics approach that revealed the enrichment of the LPS biosynthesis pathway in the BRG and GC groups. In addition, the concentrations of LPS increased significantly in the gastric juice of the BRG and GC groups, which were consistent with the results from the 16S rRNA gene sequencing and metagenome analysis. Additionally, there was no significant difference in Chao1 or Shannon index among the control, BRG, and GC groups. The changes of microbial *α* diversity in gastric cancer are not consistent in previous studies,^[^ ^42^, ^43^, ^44^, ^45^ ^]^ which might be due to the antibiotic treatment or dietary habits of the patients, or the limitation of sample size in different studies.

According to previous reports, researchers have established a model of BR using Wistar or Fischer rats through surgery and used this model to further study the relationship between BRG and gastric cancer,^[^ ^5^, ^30^ ^]^ but few studies have reported establishing a BR model in mice. Rats do not possess a gallbladder and therefore the use of BR mice which do have one was thought to be a closer pre‐clinical model. In this study, we successfully constructed a surgical model of BR in C57BL/6 J mice and used this model to further confirm the results of our clinical and cellular studies. Only 37.5% of mice in the BR surgery group developed gastric lesions after 50 weeks post‐surgery, which may be due to the long time required for bile reflux to induce gastric disease. The mice developing lesions did respond to the STAT3 inhibitor, which confirmed the results of our cell studies regarding IL‐6/JAK1/STAT3 involvement in GC development.

Bile acids could induce proliferation of rat hepatic stellate cells^[^ ^46^ ^]^ or human colon cancer cell line^[^ ^47^ ^]^ via activation of the epidermal growth factor receptor. Several studies have reported that BAs regulate the proliferation or intestinal metaplasia of gastric epithelial cells by directly interacting with their receptors, such as the Farnesoid X receptor (FXR)^[^ ^14^ ^]^ and G protein‐coupled bile acid receptor 1 (TGR5).^[^ ^36^ ^]^ In our study, we found that TDCA and LPS could promote the proliferation of gastric cells. Furthermore, the results of Western Blot showed that the protein levels of Ki67 (a proliferation marker) and Cyclin D1 (a regulator of cell cycle progression) were significantly upregulated by TDCA, LPS, and TDCA with LPS, suggesting that TDCA and LPS may promote cell proliferation by accelerating cell cycle. In the future, more mechanistic experiments should be conducted to further verify whether TDCA and LPS promote cell proliferation by accelerating the cell cycle and ultimately promote gastric carcinogenesis.

Consistently with previous studies that there was a significant increase in the potentially pathogenic bacteria in gastric cancer,^[^ ^44^, ^45^, ^48^ ^]^ such as *Prevotella* and *Veillonella*. In our study, *P. melaninogenica*, an LPS‐producing bacteria, was observed to be significantly increased in the gastric juice of BRG and GC groups. In a cohort of 276 patients with gastric cancer,^[^ ^45^ ^]^ the researchers have found that *P. melaninogenica* was significantly increased in the tumoral tissues compared to peritumoral tissues. As an oral and respiratory pathogen, *P. melaninogenica* can colonize and overgrow in the stomach of the BRG group, which may be due to a large amount of bile acids reflux into the stomach, changing the intragastric environment, such as the increase of pH value. Recently published study on Gut, 2021^[^ ^49^ ^]^ showed that gastric microbiota from patients with intestinal metaplasia and gastric cancer induced premalignant lesions in the gastric mucosa of recipient germ‐free mice after one‐year post inoculation, providing the causality of associations of human gastric microbiome in the onset of gastric cancer. The germ‐free mice model will be applied in future studies to investigate the role of the microbiota in the onset of gastric cancer.

In our study, conjugated BAs and LPS increased significantly in the stomach of the BRG and GC groups, which, in turn, promoted gastric lesions by upregulating the IL‐6/JAK1/STAT3 pathway. In the future, the number of clinical and animal samples should be expanded to verify the current findings. In addition, the correlation of conjugated BAs and gastric microbiota should also be investigated, such as the mechanism through which BAs increase the abundance of LPS‐producing bacteria. Meanwhile, GES‐1, the SV40 transformed human fetal GES, has been shown to be non‐tumorigenic in nude mice and is considered a non‐malignant cell line that can be used to mimic human gastric epithelial cells suffering from BAs reflux. Other model systems based on primary human gastric cells may serve as more realistic model systems for future studies.

## 4 Experimental Section

### Experimental Design

The main objective of this study was to investigate the changes in the BA profiles of patients with BRG and to explore the effects of refluxed BAs and microbiome on gastric carcinogenesis. Patients’ sample collection and use were approved by the ethical committee of Shanghai Jiao Tong University Affiliated Sixth People's Hospital (ethics approval number: No. 2020–097). All subjects were provided with a full explanation regarding the nature of the study, and written informed consent was obtained from each of the patients prior to starting the endoscopy (Chinese Clinical Trial Register (ChiCTR) number: ChiCTR2000035004). The subjects in the control group were those without bile reflux gastritis, gastric cancer, or the following conditions: other cancers, acute and chronic gastrointestinal diseases, acute infectious disease, receipt of any antibiotic treatment within 3 months before sample collection, regularly taking prescription Chinese or Western medicines, pregnancy, *H. pylori* treatment, history of surgery, history of taking proton pump inhibitors or histamine‐2 receptor blockers.

All animal procedures and testing were approved by the national legislation and local guidelines of the laboratory animals center at Shanghai Jiao Tong University Affiliated Sixth People's Hospital, Shanghai, China (ethics approval number: No. 2017‐0038). Sample sizes for animal studies were determined by statistical analysis of variance and on the basis of our experience with similar studies. The sample size (*n*) for each experimental group is indicated in the corresponding figure legends and was between six and eight mice per group. The number of replicates for each experiment is described in the relevant figure legends.

### Human Gastric Juices

A total of 145 patient cases who underwent an EGD examination were enrolled in the present study. Patients with a history of gastric surgery, a history of *H. pylori* eradication, pregnant patients, and patients who were judged by the attending physicians to be unsuitable for endoscopy were excluded from the study. Endoscopy was performed in the early morning in participants who had not taken any food, water, or drugs since the previous night. Gastric juice in the gastric mucous lake was aspirated as much as possible immediately before the endoscopic observation. Gastric juice was aspirated through the forceps channel of the endoscope into a recovery vessel. The clinical characteristics of the 145 subjects are listed in online Table S1, Supporting Information, and the detailed procedures of gastric juice collection is in Supporting Information.

### Animal Experiment 1

Four‐week‐old C57BL/6J male mice were purchased from Shanghai Sippr‐BK Laboratory Animal Co. Ltd. (Shanghai, China). All the mice were maintained in a specific‐pathogen‐free (SPF) environment with controlled conditions, a 12 h light/dark cycle at 20—22 °C and 45 ± 5% humidity. The mice were acclimated with a normal diet for 1 week and subsequently divided into 4 groups: Mice were orally gavaged with saline solution (control, *n* = 6), TDCA (120 mg/kg/day, *n* = 8), and LPS (0.05 mg/kg/day, *n* = 8) or TDCA (120 mg/kg/day) plus LPS (0.05 mg/kg/day) (*n* = 8) for 43 weeks.

### Animal Experiment 2

Four‐week‐old C57BL/6J male mice were purchased from Shanghai Sippr‐BK Laboratory Animal Co. Ltd. (Shanghai, China). All the mice were maintained in an SPF environment with controlled conditions, a 12 h light/dark cycle at 20—22 °C and 45 ± 5% humidity. The mice were acclimated with a normal diet for 1 week and subsequently divided into 3 groups: Mice were orally gavaged saline solution (control, *n* = 6), PM (1 × 10^9^ cfu/day, *n* = 8), and TDCA (120 mg/kg/day) plus PM (1 × 10^9^ cfu/day) (*n* = 7) respectively, for 43 weeks.

### Animal Experiment 3

Six‐week‐old C57BL/6J male mice were purchased from Shanghai Sippr‐BK Laboratory Animal Co. Ltd. (Shanghai, China) and used for constructing a BR model. All the mice were maintained in a SPF environment with controlled conditions, a 12 h light/dark cycle at 20–22 °C and 45 ± 5% humidity. The mice were acclimated with a normal diet for 1 week and subsequently divided into 3 groups: sham (*n* = 8), BR surgery (*n* = 8), and BR surgery with cryptotanshinone (*n* = 6) groups. After 24 h of fasting, a midline laparotomy incision was performed under anesthesia with 60 mg kg^−1^ sodium pentobarbital. The following procedure was performed according to previously reported methods used to construct rat reflux models.^[^ ^50^, ^51^ ^]^ Briefly, side‐to‐side anastomosis was performed between the greater curvature of the stomach and the upper jejunum located approximately 1.5 cm distally from the pylorus ring. The saline solution was injected into the peritoneal cavity to prevent adhesion and as postoperative fluid replenishment, and the wound was closed. Mice in the BR surgery with cryptotanshinone group received an intraperitoneal injection of cryptotanshinone (20 mg/kg/day). For the construction of the sham surgery group, the same pre‐ and postoperative preparations were implemented as for the reflux model. Experimental mice were allowed access to water 24 h, and to food 36 h, postoperatively, and not treated with any known carcinogens. Sequential morphological changes of the stomach of experimental mice were studied.

All the mice in the three experiments were raised with free access to normal diet and water, and their body weights and food intakes were recorded once a week. All mice were fasted overnight before being euthanized. Samples of gastric tissues and gastric contents were carefully collected and kept in liquid nitrogen and then stored at −80 °C until analysis.

### Cell Culture and Reagents

GES‐1 cells, a normal gastric epithelial cell line, purchased from Fuheng Biotechnology Co., Ltd., Shanghai, were cultured in Dulbecco's modified Eagle's medium (DMEM, Gibco). AGS, the gastric cancer cell line purchased from American Tissue Culture Collection (ATCC), was cultured in DMEM/F‐12. 10% v/v fetal bovine serum (Gibco, qualified, Australia origin) supplemented with a 1% v/v Penicillin/Streptomycin mix. All cells were incubated at 37 °C in an atmosphere containing 5% CO_2_. The reagents used in the cell experiments included TCA (J&K Scientific, 909688), TCDCA (Matrix Scientific, 100646), TDCA (J&K Scientific, 423806), TUDCA (J&K Scientific, 496672), GCA (Aladdin, G131002), GCDCA (J&K Scientific, 107563), GDCA (Aladdin, S102123), GUDCA (J&K Scientific, G0459), LPS (Sigma, L6529) and cryptotanshinone (Selleck, S2285). The ELISA kit used in the cell experiments was acquired from LEGEND MAX Human IL‐6 ELISA Kit (BioLegend, 430507).

### Cell Viability Assay

Cell viability was determined by the Cell Counting Kit‐8 (CCK‐8, Dojindo, Japan) assay. For each well in a 96‐well plate, 1500 cells were seeded with 100 µL of culture medium and treated with the conjugated BAs or LPS for 72 h. Then 10 µL of CCK‐8 solution was added to the cells and cells were incubated for 3 h at 37 °C. The reaction product was quantitatively measured according to the manufacturer's instructions.

### Cell Colony Formation Assay

For each well in a 6‐well plate, 250 cells were seeded with 3 mL of culture medium and treated with the conjugated BAs or LPS for 12 days. The number of cell colonies was counted after crystal violet staining.

### Full‐Length 16S rRNA Gene Sequencing

Total microbial genomic DNA samples were extracted using the OMEGA DNA isolation kit (Omega, D5625‐01, USA), following the manufacturer's instructions. The quantity and quality of extracted DNA were measured using a NanoDrop NC‐1000 spectrophotometer (Thermo Fisher Scientific, Waltham, MA, USA) and agarose gel electrophoresis, respectively. Polymerase chain reaction (PCR) amplification of the full‐length bacterial 16S rRNA gene was performed using the forward primer 27F (5″‐AGAGTTTGATCMTGGCTCAG‐3″) and the reverse primer 1492R (5″‐ACCTTGTTACGACTT‐3″). The Quantitative Insights Into Microbial Ecology (QIIME2) pipeline was employed to process the sequencing data at Shanghai Personal Biotechnology Co., Ltd. (Shanghai, China). The sequencing data of the full‐length 16S rRNA gene sequences are listed in online Table S2, Supporting Information. The detailed description of the pipeline of sequencing data analysis is in Supporting Information.

### Metagenomic Analysis

Total microbial genomic DNA samples were extracted using the OMEGA DNA isolation kit (Omega, D5625‐01, USA), following the manufacturer's instructions. The quantity and quality of extracted DNAs were measured using a NanoDrop ND‐1000 spectrophotometer (Thermo Fisher Scientific, Waltham, MA, USA) and agarose gel electrophoresis, respectively. The extracted microbial DNA samples were processed to construct metagenome shotgun sequencing libraries with insert sizes of 400 bp by using the Illumina TruSeq Nano DNA LT Library Preparation Kit. Each library was sequenced by the Illumina HiSeq X‐ten platform (Illumina, USA) with PE150 strategy at Personal Biotechnology Co., Ltd. (Shanghai, China). The sequencing data of the metagenomic approach are listed in online Table S3, Supporting Information. The detailed description of the pipeline of sequencing data analysis is in Supporting Information.

### *P*. *melaninogenica* Abundance Analysis

The microbial genomic DNA of gastric contents in mice was extracted using the DNeasyPowerSoil Kit (QIAGEN, Inc., Netherlands), following the manufacturer's instructions. The quantity and quality of extracted DNAs were measured using a NanoDrop ND‐1000 spectrophotometer (Thermo Fisher Scientific, Waltham, MA, USA) and agarose gel electrophoresis, respectively. The extracted microbial DNA of all samples was first diluted to the same concentration and then to construct SYBR Green‐based quantitative real‐time PCR. The extracted microbial DNA was processed to construct SYBR Green‐based quantitative real‐time PCR. The *P. melaninogenica* fraction as part of the whole bacterial population was calculated by dividing the gene copy number of *P. melaninogenica* primers^[^ ^52^ ^]^ by the total gene copy number using 16S rRNA primers. The primers used in this study are listed in online Table S4, Supporting Information.

### Luminex Multiplex Assay

The collected gastric juice of human and the gastric tissues of mice were immediately cryopreserved (−80 °C), and the concentrations of pro‐inflammatory cytokines were quantified using Luminex multiplex assay (R&D systems, LXSAHM‐06 for human and LXSAMSM‐05 for mice) according to the manufacturer's instructions.

### Bacterial Strain and Culture Condition

*P. melaninogenica* was purchased from the ATCC (ATCC, 25845) and was cultured in the ATCC Medium 2863 at 37 °C under anaerobic conditions.

### BAs Analysis

The collected gastric juices were immediately cryopreserved (−80 °C), and the BA concentrations were quantitatively determined using UPLC/TQMS (Waters, Milford, MA) according to a protocol we previously established.^[^ ^6^, ^53^, ^54^, ^55^ ^]^

### LPS Analysis

The collected human gastric juice, and mouse gastric tissues and gastric contents were immediately cryopreserved (−80 °C), and the LPS concentrations were quantitatively measured using ELISA kits (Mlbio, ml9022423 for human, ml037221‐C for mouse) according to the manufacturer's instructions.

### RT‐qPCR

Total RNA of cell samples and gastric tissues homogenized with TissueLyzer (QIAGEN) was isolated using TRIzol Reagent (Invitrogen, Life Technology, USA). The total RNA concentration was measured using a NanoDrop 2000C spectrophotometer (Thermo Fisher Scientific, Waltham, MA, USA). A purified, 500 ng sample of total RNA from each stomach sample was reverse transcribed using random hexamer primers to form the cDNA templates by employing a Prime Script RT Reagent Kit (TAKARA, Kusatsu, Japan). The qPCR primers were designed and synthesized (Sangon Biotech, Shanghai, China). The qPCR reaction mixture was set up using Power Up SYBR Green PCR Master Mix (Applied Biosystems, Thermo Fisher Scientific, USA) and the reaction was run in an ABI 7900HT Real‐Time PCR System (Applied Biosystems Instruments, Thermo Fisher Scientific, USA). All the procedures were handled following the manufacturer's instructions. The values of the target genes were normalized to glyceraldehyde 3‐phosphate dehydrogenase (GAPDH) and the relative expression level was shown as fold changes relative to the average value in the control group. The primers used in this study are listed in online Table S4, Supporting Information.

### Western Blot Analysis

Cell samples and gastric tissues were lysed with RIPA buffer (Beyotime Technology, Shanghai, China) containing 1mM PMSF (Beyotime Technology, Shanghai, China), Protease Inhibitor Cocktail Set III (Merck Millipore), and Phosphatase Inhibitor Cocktail (Sigma) in an ice bath for 30 min followed by centrifugation at 14000 g for 10 min. The supernatants were collected, and protein concentrations were measured using a BCA Protein Assay Kit (Pierce, Rockford, IL, USA). A 5 µg µL^−1^ of protein extract was combined with loading buffer (Beyotime Technology, Shanghai, China) and denatured by boiling at 100 °C for 10 min. The denatured proteins were resolved by 12% SDS‐PAGE and transferred to Immobilon‐P Transfer Membranes (Millipore Corporation, Tullagreen, IRL). The membranes were blocked with 5% BSA (Beyotime Technology, Shanghai, China) at room temperature for 1 h, incubated with primary antibodies overnight at 4 °C, and then incubated with horseradish peroxidase conjugated secondary antibodies. The bands were visualized using a SuperSignal West Pico Chemiluminescent Substrate (Thermo Scientific, Rockford, IL, USA) with a Tanon 5500 Chemiluminescent Imaging System (Tanon Science & Technology Co., Shanghai, China). The gray values of the bands were calculated using ImageJ software and were normalized to *β*‐Actin. The antibodies used for the present study and the dilutions ratios were as follows: 1:1000 for rabbit anti‐STAT3 (Cell Signaling Technology, MA, 12640S), 1:2000 for rabbit anti‐p‐STAT3 (T705) (Cell Signaling Technology, MA, 9145S), 1:1000 for rabbit anti‐JAK1 (Cell Signaling Technology, MA, 3344S), 1:1000 for rabbit anti‐p‐JAK1 (T1022) (Cell Signaling Technology, MA, 3331S), 1:1000 for rabbit anti‐Cyclin D1 (Cell Signaling Technology, MA, 2978S), 1:1000 for rabbit anti‐PCNA (Cell Signaling Technology, MA, 13110S), 1:100 for rabbit anti‐Ki67 (Abcam, UK, ab16667), and 1:1000 for rabbit anti‐*β*‐Actin (Cell Signaling Technology, MA, 4970S).

### Immunohistochemistry Analysis

For the immunohistochemistry staining of p‐STAT3 and Ki67, the gastric tissues of the C57BL/6J mice were fixed in 4% paraformaldehyde and embedded in paraffin according to the manufacturer's instructions. Then slides were deparaffinized, rehydrated, and stained with rabbit anti‐p‐STAT3 (T705) (Cell Signaling Technology, MA, 9145S, 1:200), and rabbit anti‐Ki67 (Abcam, UK, ab16667, 1:200).

### Statistical Analysis

Raw data of BAs quantification was obtained by MassLynx v4.1 and analyzed by TargetLynx v4.1 (Waters, Milford, MA). Data are expressed as mean with SEM. SPSS 26.0 (IBM SPSS, USA) and GraphPad Prism 8.0 (GraphPad Software, San Diego, USA) were used for statistical analyses and graphic generation. The sample distribution was determined using the Kolmogorov–Smirnov normality test. For statistical comparisons, one‐way analysis of variance (ANOVA) test or Kruskal‐Wallis test followed by the post‐hoc tests (Dunnett's test) for the normal or non‐normal distributed data, respectively. Spearman's rank correlation coefficients were calculated to examine the association of BAs and the pH values or the LPS‐producing bacteria. All the *p*‐values were adjusted by the false discovery rate (FDR) using the Benjamini−Hochberg method. The corrected *p*‐value of 0.05 was taken as a significance level. The 16S rRNA gene sequencing analysis was performed using QIIME2^[^ ^56^ ^]^ and R packages (v3.2.0). LEfSe was performed to detect differentially abundant taxa across groups.

## Conflict of Interest

The authors declare no conflict of interest.

## Author Contributions

W.J. conceptualized the study and designed the research. W.J. and A.Z. organized all the in vivo and in vitro studies and critical discussions of the results. S.W. performed the experiments and the overall analysis. S.W. and W.C. performed the cell experiments. A.Z., S.W., J.K., K.G., and J.Z. measured the BAs of all human samples. S.W., J.K., H.Z., X.Z., J.W., F.H., K.G., M.L., and M.Z. contributed to the animal experiments. S.W. and A.Z. drafted the manuscript and produced the figures. W.J., A.Z., C.R., J.K., X.Z., J.W., F.H., M.L., and M.Z. critically revised the manuscript.

## Supporting information

Supporting Information

Supporting Information

## Data Availability

The data that support the findings of this study are available from the corresponding author upon reasonable request.
