# Supplementary material for: Bile Acid–Microbiome Interaction Promotes Gastric Carcinogenesis
Source: Adv Sci (Weinh). 2022 Mar 14;9(16):2200263. doi: 10.1002/advs.202200263 (PMC9165488; doi:10.1002/advs.202200263)
Supplement: Supplementary file 1 — Supporting Information [file ADVS-9-2200263-s001.pdf]

ADVANCED SCIENCE Open Access

Supporting Information

for *Adv. Sci.*, DOI 10.1002/advs.202200263

Bile Acid–Microbiome Interaction Promotes Gastric Carcinogenesis

Shouli Wang, Junliang Kuang, Hongwei Zhang, Wenlian Chen, Xiaojiao Zheng, Jieyi Wang, Fengjie Huang, Kun Ge, Mengci Li, Mingliang Zhao, Cynthia Rajani, Jinshui Zhu, Aihua Zhao and Wei Jia**

Supporting Information

Bile Acid-Microbiome Interaction Promotes Gastric Carcinogenesis

Shouli Wang, Junliang Kuang, Hongwei Zhang, Wenlian Chen, Xiaojiao Zheng, Jieyi, Wang, Fengjie Huang, Kun Ge, Mengci Li, Mingliang Zhao, Cynthia Rajani, Jinshui Zhu, Aihua Zhao, Wei Jia**

S. Wang, J. Kuang, X. Zheng, J. Wang, F. Huang, K. Ge, M. Li, M. Zhao, A. Zhao, W. Jia

Center for Translational Medicine and Shanghai Key Laboratory of Diabetes Mellitus

Shanghai Jiao Tong University Affiliated Sixth People's Hospital

Shanghai 200233, China.

E-mail: zhah@sjtu.edu.cn; weijia1@hkbu.edu.hk

W. Jia

School of Chinese Medicine

Hong Kong Baptist University

Kowloon Tong, Hong Kong, China

H. Zhang

Department of Metabolic & Bariatric Surgery

Shanghai Jiao Tong University Affiliated Sixth People's Hospital

Shanghai 200233, China

W. Chen

Cancer Institute, Longhua Hospital

Shanghai University of Traditional Chinese Medicine

Shanghai 200233, China

W. Jia, C. Rajani

University of Hawaii Cancer Center
Honolulu, HI 96813, USA
J. Zhu
Department of Gastroenterology
Shanghai Jiao Tong University Affiliated Sixth People's Hospital
Shanghai 200233, China

Supplementary methods

Human gastric juices

No gastric mucolytic agents, foaming mucus removers, or gastrointestinal motility depressants were used, and subjects were asked to spit out all the local anesthetic used for pharyngeal anesthesia. To prevent postural reflux of duodenal fluid into the stomach, the subjects remained seated until just before the start of the endoscopy. In addition, when a lesion with hemorrhage (i.e., peptic ulcer, gastric cancer with ulceration) was found or if the gastric mucosa was injured and bleeding during aspiration of gastric juice, the subject was excluded from the study to avoid the confounding effects of BAs in the blood on the measurement results.

Full-length 16S rRNA gene sequencing

The extracted DNA was amplified with two-step PCR, with sample-specific 16-bp barcodes incorporated into the forward and reverse primers for multiplex sequencing in the second PCR step. Both of the two steps of the PCR components contained 5 μl of Q5 reaction buffer (5×), 5 μl of Q5 High-Fidelity GC buffer (5×), 0.25 μl of Q5 High-Fidelity DNA Polymerase (5U/μl), 2 μl (2.5 mM) of dNTPs, 1 μl (10 uM) of each Forward and Reverse primer, 2 μl of DNA

Template, and 8.75 µl of ddH2O. Thermal cycling consisted of initial denaturation at 98°C for 2 min, followed by 25/10 cycles (for first and second amplification step, respectively) consisting of denaturation at 98°C for 30 s, annealing at 55°C for 30 s, and extension at 72°C for 90 s, with a final extension of 5 min at 72°C. A total of PCR amplicons were purified using Agencourt AMPure Beads (Beckman Coulter, Indianapolis, IN) and quantified using the PicoGreen dsDNA Assay Kit (Invitrogen, Carlsbad, CA, USA). After the individual quantification step, amplicons were pooled in equal amounts, and Single Molecule Real Time (SMRT) sequencing technology was performed using the PacBio Sequel platform at Shanghai Personal Biotechnology Co., Ltd (Shanghai, China). In order to decrease the sequencing error rate, PacBio circular consensus sequencing (CCS) reads were derived from the multiple alignments of sub-reads. In CCS, the DNA polymerase reads a ligated circular DNA template multiple times, which can effectively generate a consensus sequence from multiple reads of a single molecule. Raw sequences were initially processed through the PacBio SMRT Link portal. Sequences were filtered for a minimum of 3 passes, and a minimum predicted accuracy of 99% (minfullpass =3, minPredictedAccuracy =99). The predicted accuracy of 99%, which is defined as the threshold below which a CCS is considered as noise. The files generated by the PacBio platform were then used for amplicon size trimming to remove sequences with length longer than 2000 bp.

Briefly, raw sequence data were demultiplexed using the demux plugin following by primers cutting with Cutadapt plugin [1]. Sequences were then quality filtered, denoised, merged and chimera removed using the DADA2 plugin [2]. Non-singleton amplicon sequence variants (ASVs) were aligned with mafft [3] and used to construct a phylogeny with fasttree2 [4]. Taxonomy was assigned to ASVs using the classify-sklearn naïve Bayes taxonomy classifier in feature-classifier plugin [5] against the SILVA Release 132 Database.

Metagenomic analysis

Raw sequencing reads were processed to obtain quality-filtered reads for further analysis. First, the sequencing adapters were removed from sequencing reads using Cutadapt (v1.2.1) [1]. Secondly, low quality reads were trimmed by using a sliding-window algorithm. Thirdly, reads were aligned to the host genome using BWA [6] (http://bio-bwa.sourceforge.net/) to remove host contamination. Once quality-filtered reads were obtained, they were *de novo* assembled to construct the metagenome for each sample by Megahit (v1.1.2) [7]. All coding regions (CDS) of metagenomic scaffolds longer than 300 bp were predicted by MetaGeneMark [8].

CDS sequences of all samples were clustered by CD-HIT [9] at 90% protein sequence identity, to obtain a non-redundant gene catalog. Gene abundance in each sample was estimated by soap.coverage (http://soap.genomics.org.cn/) based on the number of aligned reads. The lowest common ancestor taxonomy of the non-redundant genes was obtained by aligning them against the NCBI-NT database by BLASTN (e value <0.001). Similarly, the functional profiles of the non-redundant genes were obtained by annotated against the KEGG databases, by using the DIAMOND [10] alignment algorithm.

Supplementary figures

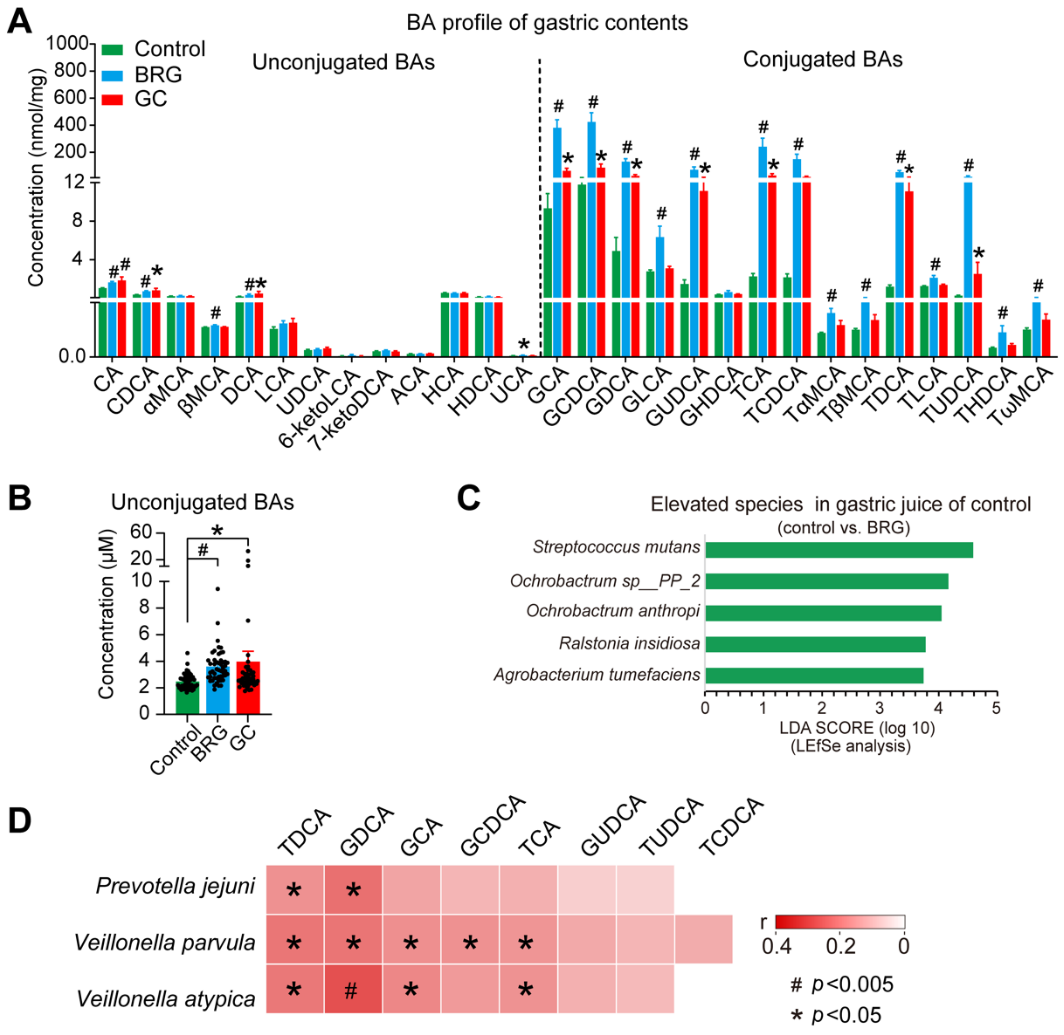

Figure S1.

The BA profiles and composition of gastric microbiota in human gastric juice.

(A) BA profiles of human gastric juice of control, BRG, and GC groups. (B) Concentrations of unconjugated BAs in human gastric juice of control, BRG, and GC groups. All groups were compared to the control group. (C) Elevated species in gastric juice of control group based on the LEfSe analysis between BRG and control groups. (D) Heatmaps of Spearman correlation

coefficients of 8 representative conjugated BAs and representative LPS-producing bacteria in human gastric juice. The color of the cells indicates the Spearman correlation coefficients (r). Data are shown as mean with SEM. Differences between groups were assessed using the Kruskal-Wallis, # $p < 0.005$, * $p < 0.05$.

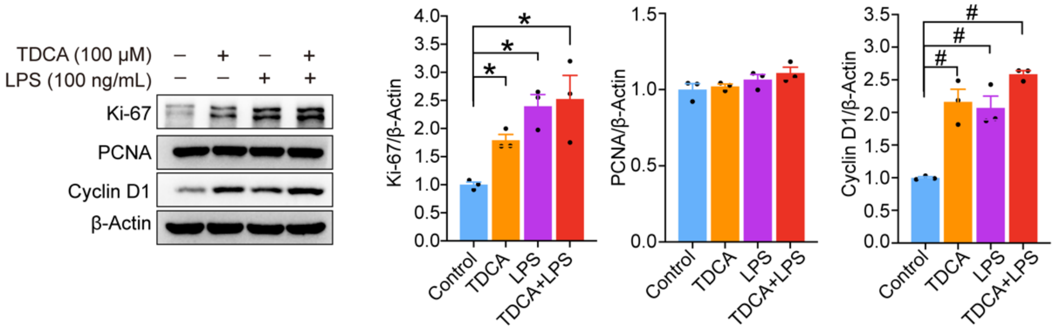

Figure S2.

Western blot analysis showing the protein levels of Ki-67, PCNA and Cyclin D1 in control, TDCA, LPS and TDCA+LPS groups on GES-1 cells. Data are shown as mean with SEM. Differences between groups were assessed using the one-way ANOVA test # $p < 0.005$, * $p < 0.05$.

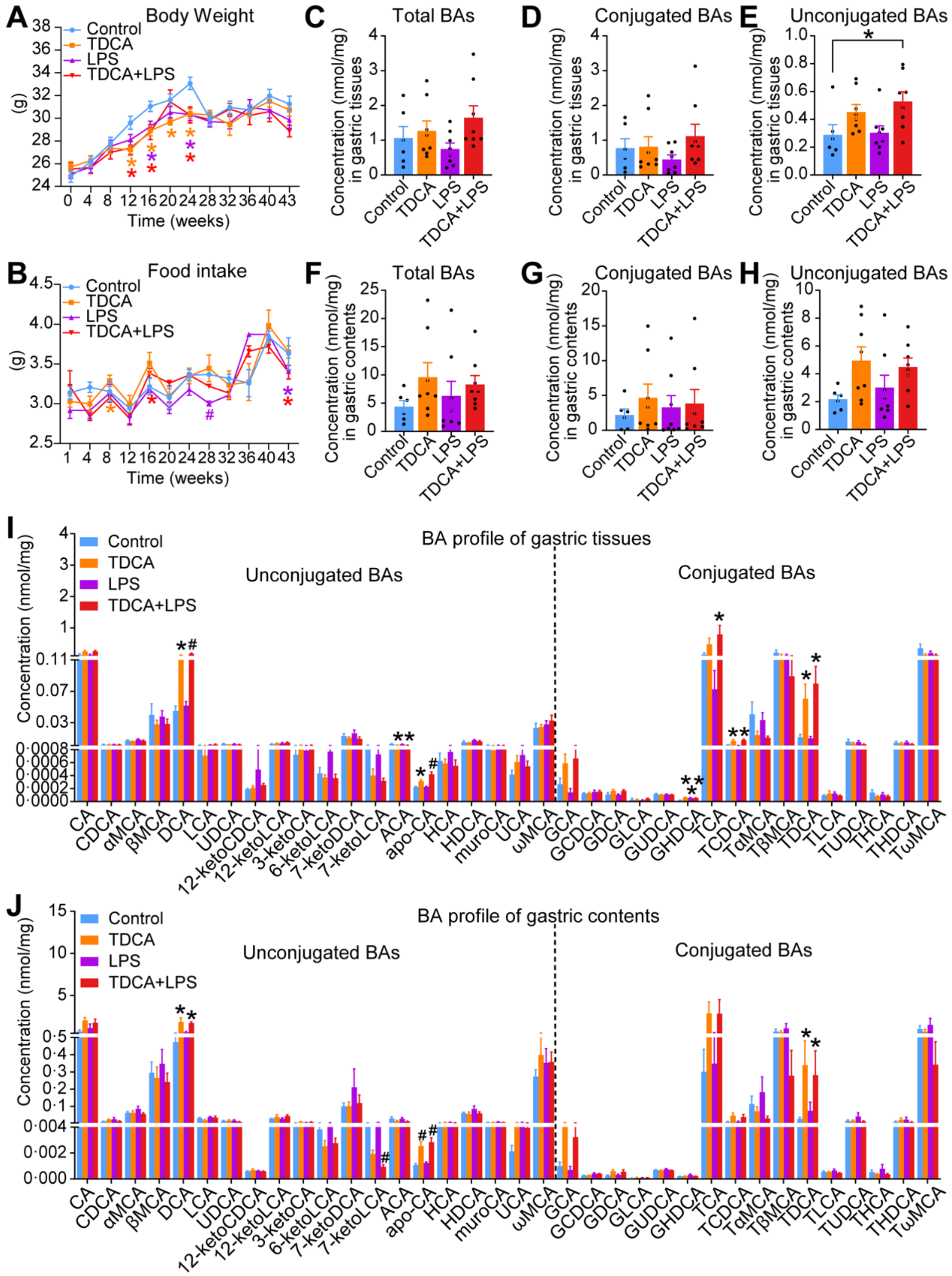

Figure S3.

The body weight, food intake and BA profiles of mice treated with TDCA, LPS and TDCA+LPS. (A) The body weight of mice from control, TDCA, LPS and TDCA+LPS groups.

(B) The food intake of mice from control, TDCA, LPS and TDCA+LPS groups. (C) Concentrations of total BAs in gastric tissues from control, TDCA, LPS and TDCA+LPS groups. (D) Concentrations of conjugated BAs in gastric tissues from control, TDCA, LPS and TDCA+LPS groups. (E) Concentrations of unconjugated BAs in gastric tissues from control, TDCA, LPS and TDCA+LPS groups. (F) Concentrations of total BAs in gastric contents from control, TDCA, LPS and TDCA+LPS groups. (G) Concentrations of conjugated BAs in gastric contents from control, TDCA, LPS and TDCA+LPS groups. (H) Concentrations of unconjugated BAs in gastric contents from control, TDCA, LPS and TDCA+LPS groups. (I) BA profiles in gastric tissues from control, TDCA, LPS and TDCA+LPS groups and all groups were compared to the control group. (J) BA profiles in gastric contents from control, TDCA, LPS and TDCA+LPS groups and all groups were compared to the control group. Data are shown as mean with SEM. Differences between groups were assessed using the Kruskal-Wallis test, # $p < 0.005$, * $p < 0.05$.

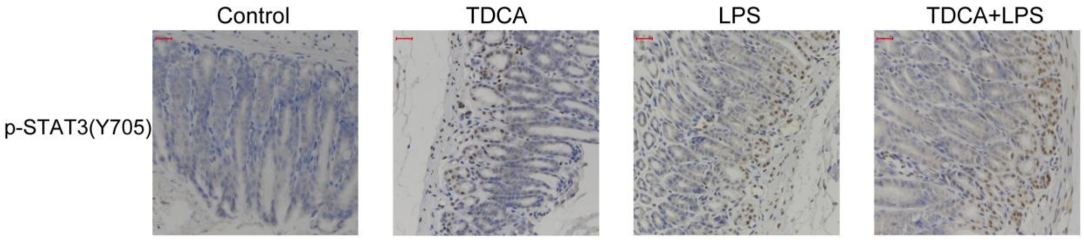

Figure S4.

Representative images of immunohistochemistry for p-STAT3 of gastric tissues from control, TDCA, LPS and TDCA+LPS groups, bars, 25 µm.

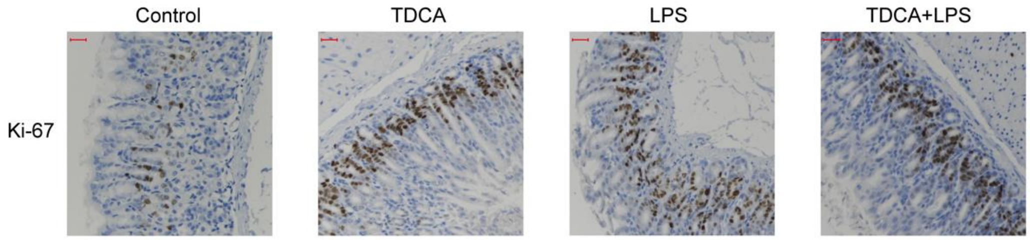

Figure S5.

Representative images of immunohistochemistry for Ki-67 of gastric tissues from control, TDCA, LPS and TDCA+LPS groups, bars, 25 μm.

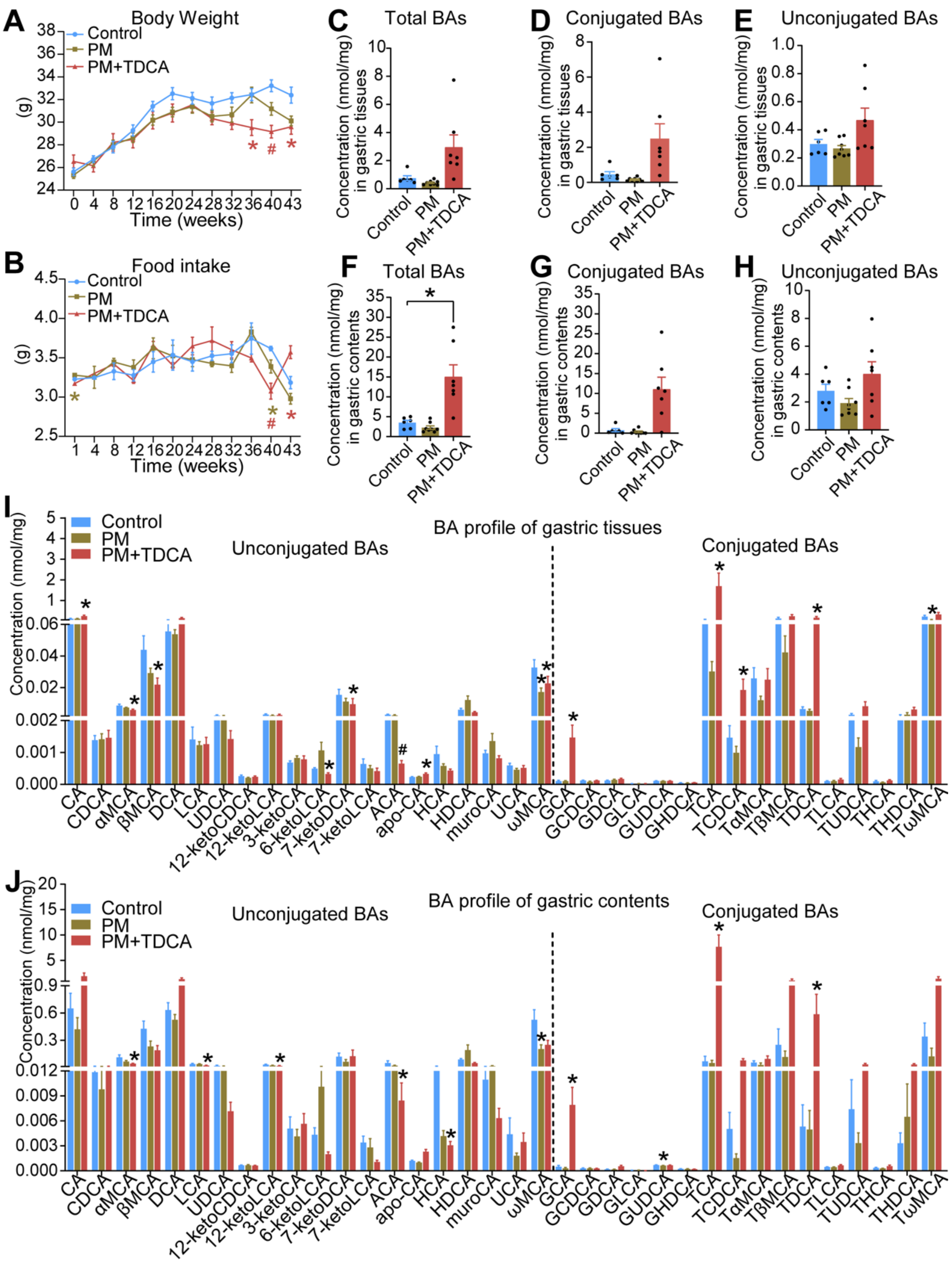

Figure S6.

The body weight, food intake and BA profiles of mice treated with PM and PM+TDCA. (A) The body weight of mice from control, PM and PM+TDCA groups. (B) The food intake of mice

from control, PM and PM+TDCA groups. (C) Concentrations of total BAs in gastric tissues from control, PM and PM+TDCA groups. (D) Concentrations of conjugated BAs in gastric tissues from control, PM and PM+TDCA groups. (E) Concentrations of unconjugated BAs in gastric tissues from control, PM and PM+TDCA groups. (F) Concentrations of total BAs in gastric contents from control, PM and PM+TDCA groups. (G) Concentrations of conjugated BAs in gastric contents from control, PM and PM+TDCA groups. (H) Concentrations of unconjugated BAs in gastric contents from control, PM and PM+TDCA groups. (I) BA profiles in gastric tissues from control, PM and PM+TDCA groups and all groups were compared to the control group. (J) BA profiles in gastric contents from control, PM and PM+TDCA groups and all groups were compared to the control group. Data are shown as mean with SEM. Differences between groups were assessed using the Kruskal-Wallis test, # $p < 0.005$, * $p < 0.05$.

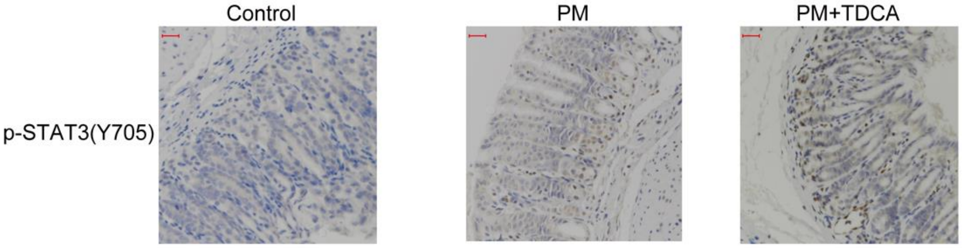

Figure S7.

Representative images of immunohistochemistry for p-STAT3 of gastric tissues from PM and PM+TDCA groups, bars, 25 µm.

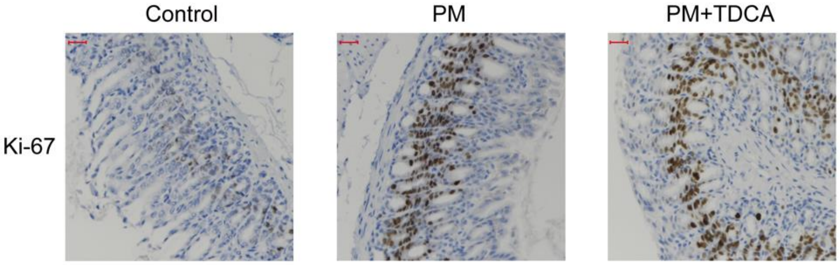

Figure S8.

Representative images of immunohistochemistry for Ki-67 of gastric tissues from PM and PM+TDCA groups, bars, 25 μm.

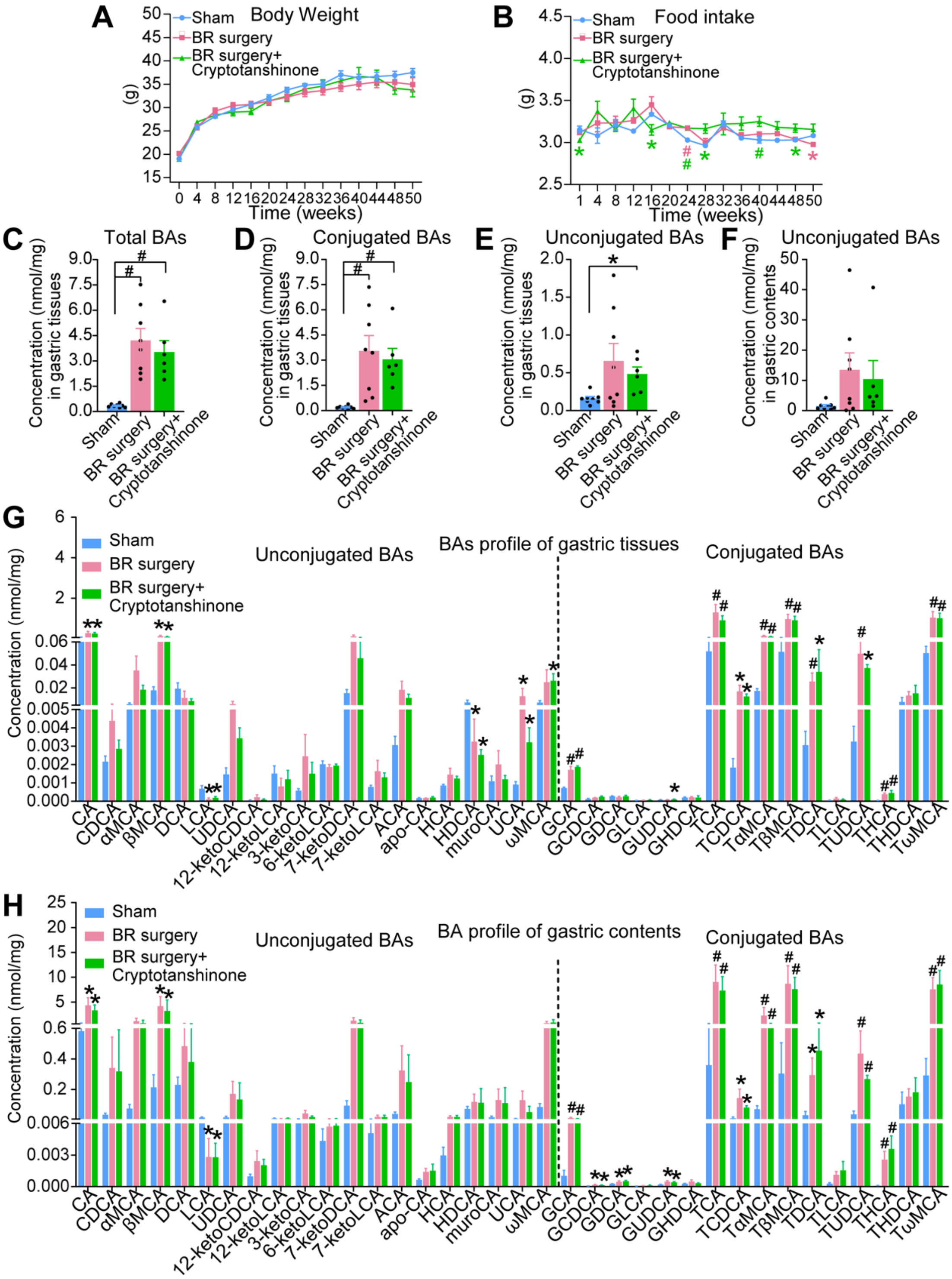

Figure S9.

The body weight, food intake and BA profiles of mice treated with BR surgery and BR surgery+cryptotanshinone. (A) The body weight of mice from sham, BR surgery and BR surgery+cryptotanshinone groups. (B) The food intake of mice from sham, BR surgery and BR surgery+cryptotanshinone groups. (C) Concentrations of total BAs in gastric tissues from sham, BR surgery and BR surgery+cryptotanshinone groups. (D) Concentrations of conjugated BAs in gastric tissues from sham, BR surgery and BR surgery+cryptotanshinone groups. (E) Concentrations of unconjugated BAs in gastric tissues from sham, BR surgery and BR surgery+cryptotanshinone groups. (F) Concentrations of unconjugated BAs in gastric contents from sham, BR surgery and BR surgery+cryptotanshinone groups. (G) BA profiles in gastric tissues from sham, BR surgery and BR surgery+cryptotanshinone groups and all groups were compared to the sham group. (H) BA profiles in gastric contents from sham, BR surgery and BR surgery+cryptotanshinone groups and all groups were compared to the sham group. Data are shown as mean with SEM. Differences between groups were assessed using the Kruskal-Wallis test, # $p < 0.005$, * $p < 0.05$.

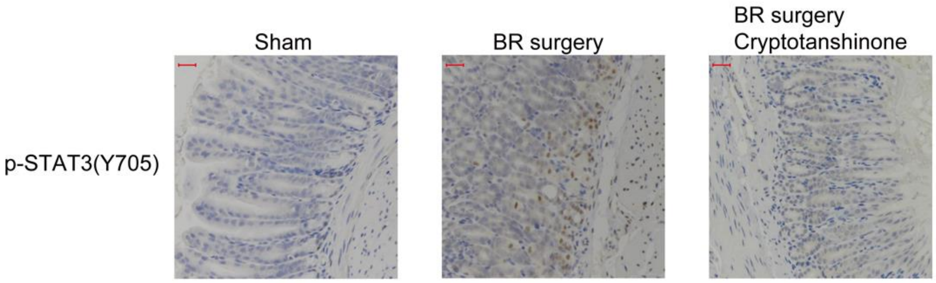

Figure S10.

Representative images of immunohistochemistry for p-STAT3 of gastric tissues from sham, BR surgery and BR surgery+cryptotanshinone groups, bars, 25 µm.

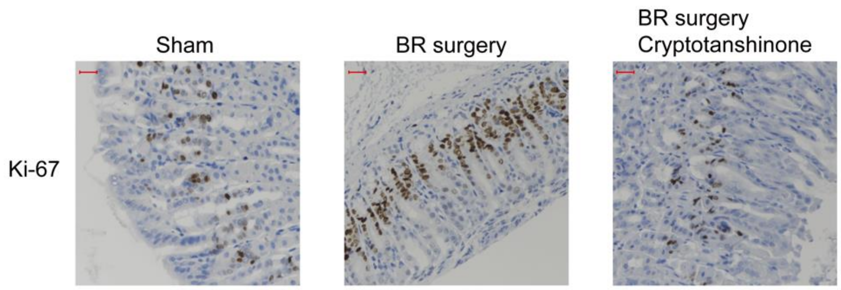

Figure S11.

Representative images of immunohistochemistry for Ki-67 of gastric tissues from sham, BR surgery and BR surgery+cryptotanshinone groups, bars, 25 μm.

References

[1] M. Martin, *Embnet Journal.* **2011**, *17*.

[2] B. J. Callahan, P. J. McMurdie, M. J. Rosen, A. W. Han, A. J. Johnson, S. P. Holmes, *Nat Methods.* **2016**, *13*, 581.

[3] K. Katoh, K. Misawa, K. Kuma, T. Miyata, *Nucleic Acids Res.* **2002**, *30*, 3059.

[4] M. N. Price, P. S. Dehal, A. P. Arkin, *Mol Biol Evol.* **2009**, *26*, 1641.

[5] N. A. Bokulich, B. D. Kaehler, J. R. Rideout, M. Dillon, E. Bolyen, R. Knight, G. A. Huttley, J. Gregory Caporaso, *Microbiome.* **2018**, *6*, 90.

[6] H. Li, R. Durbin, *Bioinformatics.* **2009**, *25*, 1754.

[7] D. Li, C. M. Liu, R. Luo, K. Sadakane, T. W. Lam, *Bioinformatics.* **2015**, *31*, 1674.

[8] W. Zhu, A. Lomsadze, M. Borodovsky, *Nucleic Acids Res.* **2010**, *38*, e132.

[9] L. Fu, B. Niu, Z. Zhu, S. Wu, W. Li, *Bioinformatics.* **2012**, *28*, 3150.

[10] B. Buchfink, C. Xie, D. H. Huson, *Nat Methods.* **2015**, *12*, 59.
